# A “2-in-1” Bioanalytical System Based on Nanocomposite Conductive Polymers for Early Detection of Surface Water Pollution

**DOI:** 10.3390/polym16101431

**Published:** 2024-05-17

**Authors:** Anna S. Kharkova, Anastasia S. Medvedeva, Lyubov S. Kuznetsova, Maria M. Gertsen, Vladimir V. Kolesov, Vyacheslav A. Arlyapov, Anatoly N. Reshetilov

**Affiliations:** 1 The Research Center «BioChemTech», Tula State University, 300012 Tula, Russia; anyuta_zaytseva@mail.ru (A.S.K.); ilyuhina.nastya@mail.ru (A.S.M.); l.s.latunina@gmail.com (L.S.K.); 2 Laboratory of Soil Chemistry and Ecology, Tula State Lev Tolstoy Pedagogical University, 300026 Tula, Russia; gertsen@tsput.ru; 3 Kotelnikov Institute of Radioengineering and Electronics (IRE) of Russian Academy of Sciences, 111250 Moscow, Russia; kvv@cplire.ru; 4 Federal Research Center «Pushchino Scientific Center for Biological Research of the Russian Academy of Sciences», G.K. Skryabin Institute of Biochemistry and Physiology of Microorganisms, Russian Academy of Sciences, 142290 Pushchino, Russia; anatol@ibpm.pushchino.ru

**Keywords:** mediator biosensor, water toxicity, early-warning instrument, redox-active polymer, biochemical oxygen demand (BOD)

## Abstract

This work proposes an approach to the formation of receptor elements for the rapid diagnosis of the state of surface waters according to two indicators: the biochemical oxygen demand (BOD) index and toxicity. Associations among microorganisms based on the bacteria *P. yeei* and yeast *S. cerevisiae*, as well as associations of the yeasts *O. polymorpha* and *B. adeninivorans*, were formed to evaluate these indicators, respectively. The use of nanocomposite electrically conductive materials based on carbon nanotubes, biocompatible natural polymers—chitosan and bovine serum albumin cross-linked with ferrocenecarboxaldehyde, neutral red, safranin, and phenosafranin—has made it possible to expand the analytical capabilities of receptor systems. Redox polymers were studied by IR spectroscopy and Raman spectroscopy, the contents of electroactive components were determined by atomic absorption spectroscopy, and electrochemical properties were studied by electrochemical impedance and cyclic voltammetry methods. Based on the proposed kinetic approach to modeling individual stages of bioelectrochemical processes, the chitosan–neutral red/CNT composite was chosen to immobilize the yeast association between *O. polymorpha* (k_s_ = 370 ± 20 L/g × s) and *B. adeninivorans* (320 ± 30 L/g × s), and a bovine serum albumin (BSA)–neutral composite was chosen to immobilize the association between the yeast *S. cerevisiae* (k_s_ = 130 ± 10 L/g × s) and the bacteria *P. yeei* red/CNT (170 ± 30 L/g × s). After optimizing the composition of the receptor systems, it was shown that the use of nanocomposite materials together with associations among microorganisms makes it possible to determine BOD with high sensitivity (with a lower limit of 0.6 mg/dm^3^) and detect the presence of a wide range of toxicants of both organic and inorganic origin. Both receptor elements were tested on water samples, showing a high correlation between the results of biosensor analysis of BOD and toxicity and the results of standard analytical methods. The results obtained show broad prospects for creating sensitive and portable bioelectrochemical sensors for the early warning of environmentally hazardous situations based on associations among microorganisms and nanocomposite materials.

## 1. Introduction

Monitoring wastewater treatment and controlling natural water experiencing high anthropogenic load from enterprises are priority tasks in preventing environmentally hazardous situations [1,2,3]. Universal analytical systems for the real-time measurements of several chemical contents allow tracking the spread of pollution, as well as quickly and accurately answering questions about water quality in a particular region. Now, to fully monitor natural water quality in environmental laboratories, there is a significant amount of analytical equipment; this equipment is based on various physicochemical methods and imposes various requirements for sample preparations and employee qualifications.

The most important integral indicators of water pollution are biochemical oxygen demand (BOD) and toxicity [4,5]. Laboratory models of biotoxicity analyzers are based on different test organisms such as fish, daphnia, algae, ciliates, duckweed, animal cells, and certain types of bacteria [6,7]. For most biotesting methods, the main disadvantage is the long duration of the analysis. Microorganism applications that have physiological reactions to pollutants that are similar to higher organisms for biosensor development make it possible to carry out rapid analysis. Commercial analyzers, Microtox (Modern Water, York, UK) and Biotox-10M (LLC STC “Econ”, Moscow, Russia), are examples of the successful use of microorganisms to assess toxicity. Both devices are based on bioluminescent bacteria that are sensitive to organic substances and less sensitive to heavy metals [8]. The BOD index quantitatively indicates the ability of wastewater to decrease the oxygen content. The standard method is based on incubating a sample for at least 5 days. The danger of a 5-day analysis is that it does not allow for assessing the real threat posed by bio-oxidizable organic impurities in effluent disposed into natural water after an accident at a wastewater treatment plant; therefore, it is impossible to take steps to eliminate the consequences until the standard analysis is completed. The kinetic approach to BOD assessing, implemented in microbial biosensors, can reduce the analysis time to several minutes. To date, a significant number of laboratory models and several industrially produced biosensor BOD analyzers are known (now, the average cost of a commercial BOD biosensor is about USD 30,000). Reviews [9,10,11] have summarized information on BOD sensors developed to date, making it possible to determine BOD in the average range of 5–300 mg/L in about 5–60 min. Current problems regarding the development of BOD sensors include increasing the sensitivity of the analysis, increasing the long-term stability, simplifying maintenance requirements, and the insufficient resistance of the used microorganism cultures to heavy metals and various toxic substances [12,13].

Most commercially available devices for environmental monitoring are based on the electrochemical determination of the rate of formation of reaction products (first-generation biosensors) [12,14]. Later models of biosensors are based on compounds that can be reversibly oxidized/reduced and interact with the active center of the enzyme—electron transport mediators (second-generation biosensors) [15]. A more modern approach to the development of mediator biosensor systems, which enhances the sensitivity of the method, reduces the analysis time and enables miniaturization, is the immobilization of biomaterial into redox-active polymers. Their complex structures consist of molecules of electroactive compounds (electron transport mediators) covalently bonded to a polymer base, which may or may not be electrically conductive. This approach ensures high sensitivity, a short analysis time, and the potential for miniaturization of biosensor systems [16,17,18,19,20]. The redox-active polymer has a dual function. Firstly, it holds the biomaterial in place due to a network structure formation with pore sizes that support microorganism immobilization. Secondly, due to a covalently bound mediator in the polymer structure, the matrix provides electron transport from the active centers of microorganism enzymes onto the electrode. Thus, biosensors based on redox-active polymers become reagent-free, and additional reagents for analysis are not required.

Carbon nanomaterials, such as single-walled and multi-walled carbon nanotubes, nanodots, and graphene, are added to the “biomaterial—redox-active polymer” receptor system to increase sensitivity [13]. After nanoparticles are introduced into the receptor element, the conductivity of the system increases significantly. In addition, at low yields of polymer modification by the redox-active compound, there are not enough electroactive species to quickly transfer electrons to the electrode; the added nanomaterials reduce the kinetic limitation by increasing the electrode’s effective surface area and providing conductivity between covalently bonded redox particles, between which, electron transfer does not occur.

**Figure 1:**
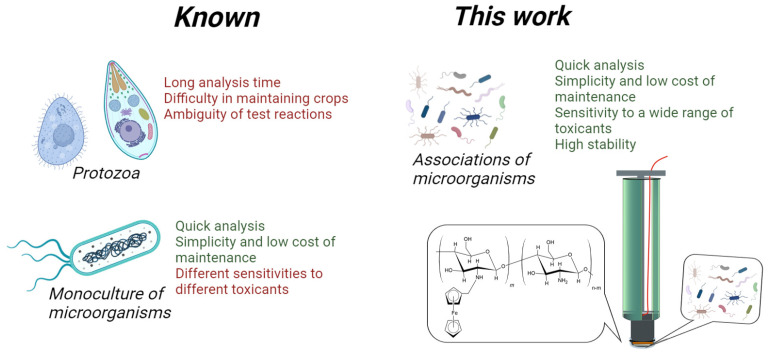
The biosensor formation approach used in this study.

The use of redox-active polymers and their nanocomposites for the amperometric assessment of BOD and toxicity has attracted much attention due to their ease of miniaturization, reliability, and low cost [9,21,22]. The biosensor current is generated by redox-active particles of the polymer; they transfer electrons from the biomaterial to the electrode. When a toxicant is added to this system, the respiratory activity of the microbe is inhibited and, as a result, the recorded signal decreases. The kinetic approach to assessing the degree of reduction of respiratory activity in the presence of a toxicant makes it possible to reduce the time of biotesting to several minutes. In contrast to luminescent biosensors, the amperometric type of detection using redox-active polymers allows for the use of a large number of individual microorganisms and the formation of bioreceptor-based consortia that are sensitive to both organic toxicants and heavy metals. Thus, reference [23] proposes the use of *E. coli* bacteria immobilized in a chitosan-based polymer to assess the toxicity of natural water. The glassy carbon electrode is modified with carbon nanodots—a type of discrete and quasi-spherical nanoparticles with good conductivity and biocompatibility. The sensor was tested on 18 natural water samples and showed a good correlation with the results of the standard luminescent method (R^2^ = 0.9827). Today, microbial biosensors are less sensitive compared to standard test objects, for example, daphnia and duckweed [8] (Figure 1), so the search for the most sensitive receptor systems based on microorganisms is crucial; known experiences are used to form stable associations among microorganisms in order to increase sensitivity to a wide range of toxicants. The application of redox-active polymers for BOD biosensor development has been reported in several works [13,20]. Reference [13] proposes a more effective method for electron transfer from yeast cells to an electrode, using a two-mediator system based on polythionine and neutral red. Single-walled carbon nanotubes are used to amplify the analytical signal, making it possible to estimate BOD at lower concentrations, such as 0.4 mg/L.

A large number of publications regularly published on this topic indicate that no characteristics have yet been obtained that could stop the process of further research into biosensor systems to determine biochemical oxygen consumption and toxicity. Therefore, in this area, there are vast opportunities for the use of electrodes modified with composite materials and microbial cells (Figure 1). The novelty of this work lies in connecting the association of microorganisms with a physicochemical detector—an electrode—via a nanocomposite that measures two indicators of the quality of aquatic environments: the index of biochemical oxygen demand and toxicity. It should be noted that this research is more actual for toxicity assessment, because nowadays, for analysis, there is a need to add a mediator into the measuring cell. Fixing the mediator spices into the bio-conductive layer with the nanocomposite would significantly increase the quality of the analysis. This paper describes a method that combines composite materials “redox-active polymers—carbon nanoparticles” with microbial associations, taking into account the rate of electron transfer at each stage of bioelectrocatalysis. This allows one to develop receptor systems for the rapid monitoring of several environmental parameters.

## 2. Materials and Methods

### 2.1. Reagents and Materials

A dialysis membrane (Roth, Dautphetal, Germany) with a transmission limit of 14 kDa and graphite powder (Fluka, Berlin, Germany) were used alongside mineral oil (Helikon, Moscow, Russia) to prepare a working graphite paste electrode. The polymers used included low molecular weight chitosan (average molecular weight 50–190 kDa; (Sigma-Aldrich, St. Louis, MO, USA) and bovine serum albumin (BSA) (molecular weight 66.5 kDa; Sigma-Aldrich). Electron transfer mediators used were ferrocene (Sigma-Aldrich, St. Louis, MO, USA), ferrocenecarboxaldehyde (Sigma-Aldrich, St. Louis, MO, USA), neutral red (Diaem, Moscow, Russia), safranin O (Diaem, Moscow, Russia), and phenosafranin (Diaem, Moscow, Russia). Multi-walled carbon nanotubes (MWCNTs) with different functional groups were used as nanomaterials, including carboxylated MWCNTs (0.1–1.0 mmol/g of COOH groups; TAUNIT, Tomsk, Russia) and amidated MWCNTs (0.1−0.6 mmol/g of CONH_2_; TAUNIT, Tomsk, Russia). D-glucose (Panreac, Barcelona, Spain), peptone (Condra, Barcelona, Spain), yeast extract (Helikon, Moscow, Russia), dipotassium phosphate (Diaem, Moscow, Russia), tryptone (Panreac, Barcelona, Spain), sodium chloride (Diaem, Moscow, Russia), and agar–agar (Panreac, Barcelona, Spain) were used for growing microorganisms. All salts used in the Tamiya medium for growing Chlorella vulgaris were chemically pure (Diaem, Moscow, Russia): KH_2_PO_4_, MgSO_4_·7H_2_O, KNO_3_, FeC_6_H_5_O_7_, H_3_BO_3_, MnCl_2_·4H_2_O, ZnSO_4_·5H_2_O, MoO_3_, NH_4_VO_3_, Co(NO_3_)_2_·6H_2_O.

### 2.2. Microorganisms

The yeast strains *Blastobotrys adeninivorans* VKM Y-2677 (*B. adeninivorans*), *Saccharomyces cerevisiae* VKM Y-1173 (*S. cerevisiae*), and *Ogateae polymorpha* VKM Y-2559 (*O. polymorpha*) were provided by the All-Russian Collection of Microorganisms at the Federal Research Center, “Pushchino Scientific Center for Biological Research of the Russian Academy of Sciences” (Russia). *Escherichia coli* K-802 (*E. coli*) bacteria were provided by the Laboratory of Plasmid Biology at the Institute of Biochemistry and Physiology of Microorganisms, part of the Federal Research Center, “Pushchino Scientific Center for Biological Research of the Russian Academy of Sciences”. The bacteria *Paracoccus yeei* VKM B-3302 (*P. yeei*) were previously isolated from activated sludge.

### 2.3. Cultivation of Microorganisms

Cell microorganisms were cultured using an ES-20/60 orbital shaker incubator (BioSan, Riga, Latvia), TG16WS centrifuges (Polycom, Moscow, Russia), and MiniSpin Plus (Eppendorf, Moscow, Russia). The biomass was maintained in microtubes at −25 °C. All cultured media were sterilized using a VP-01/75 autoclave (TPMEI, Moscow, Russia).

*P. yeei* and *E. coli* bacteria were grown in an LB medium. The composition of the liquid medium was tryptone—10 g/dm^3^, yeast extract—5 g/dm^3^, and sodium chloride—10 g/dm^3^. The cell growth medium was sterilized by an autoclave pressure of 1.15 atm within 45 min. Cells were aerobically grown in 750 cm^3^ shaking flasks for 20 to 24 h at a temperature of 29 °C. Then, the biomass was centrifuged for 10 min at room temperature at 6000× *g* and then washed from the culture media with 20 mM phosphate buffer at a pH of 6.8. Settled cells were then resuspended in fresh portions of the buffer, distributed into aliquots, and centrifuged for 10 min at 6000× *g*. The washed biomass was weighed and stored at –25 °C.

Yeasts *S. cerevisiae* and *B. adeninivorans* were cultured in a medium containing the following components: glucose—6.25 g/dm^3^, peptone—6.25 g/dm^3^, yeast extract—3.75 g/dm^3^, and K_2_HPO_4_—0.35 g/dm^3^. The cell culture medium was sterilized by autoclaving under a pressure of 1.1 atmospheres for 45 min. The cells were aerobically grown in shaking flasks for 18 to 20 h at a temperature of 29 °C. The biomass was then centrifuged for 10 min at room temperature at 6000× *g* and the supernatant was discarded. The cells were washed with 20 mM phosphate buffer at pH 6.8, and the cell pellets were transferred to fresh buffer and distributed into equal portions. The pellets were centrifuged for 10 min at 6000× *g*. The washed biomass was then weighed and stored at −25 °C in microtubes.

The yeast *O. polymorpha* was cultured in a medium containing the following components: glycerol—1%; NH_4_H_2_PO_4_—0.39%; NH_4_SO_4_—0.25%; trace elements—0.1%; yeast extract—0.05%; magnesium sulfate—0.04%; potassium dihydrogen phosphate—0.09%, and leucine—0.017%. The cell growth medium was sterilized using autoclaving at 1-atmosphere pressure for 45 min. Cells were aerobically grown in 750 mL shaking flasks for 36 h at 28 °C. After 36 h, the resulting biomass was harvested by centrifugation at 9000× *g* for 15 min. The precipitate was washed with 20 mM phosphate buffer at pH 7.2, and the settled cells were resuspended in fresh portions of buffer and distributed into aliquots. The pellets were centrifuged for 5 min at 6000× *g*. After pelleting, the washed biomass was weighed and stored at −25 °C in microtubes.

### 2.4. Association Stability Assessment

To determine the stability of formed associations, operating biosensor conditions were simulated. For toxicity assessment, the biomass of two kinds of microorganisms with a total titer of 330 mg/mL (1:1) in a volume of 200 μL was added to a microtube. Then, a potassium–sodium phosphate buffer solution with a pH of 6.8 and a glucose solution containing a model toxicant—copper (II) ions—were added. This mixture was incubated for 5 min. It was washed three times with buffer solution and the number of cells was determined using the Koch method. Several successively diluted suspensions of microbial cocultures (degree of dilution, 10^−6^) were prepared to create separate colonies. The suspensions were inoculated using the surface method, from the three last dilutions (four parallel inoculations), and the colonies were counted after 7 days of incubation.

For the BOD determination, the biomass of two kinds of microorganisms with a total titration of 330 mg/mL (1:1) in a volume of 200 μL was added to the microtube. Then, a potassium–sodium phosphate buffer solution with a pH of 6.8 and a glucose–glutamic acid solution was added (1:1). The total concentration of the solution was 3 g/L in a volume of 50 μL. This mixture was incubated for 5 min and then washed three times with the buffer solution. Several successively diluted suspensions of microbial cocultures (degree of dilution, 10^−6^) were prepared to create separate colonies. The suspensions were inoculated using the surface method, from the three last dilutions (four parallel inoculations), and the colonies were counted after 7 days of incubation.

### 2.5. Formation of Working Electrodes

Formation of an unmodified graphite-paste electrode (GP electrode).

The working electrode was created by filling a plastic tube with a surface area of 6.3 mm^2^. The graphite powder and mineral oil were mixed in a paste, with 100 mg of the graphite powder and 40 µL of mineral oil.

Formation of a ferrocene-modified graphite-paste electrode (FC electrode).

The ferrocene-modified electrode was formed as follows: 90 mg of graphite powder, 40 μL of paraffin oil, and a solution of ferrocene in acetone (10 mg of ferrocene in 500 μL of acetone) was mixed. The resulting paste was filled into a plastic tube.

Formation of a graphite-paste electrode modified with a redox-active polymer based on chitosan (CHIT-NR; CHIT-SFR; CHIT-FEN electrodes).

**Figure 2:**
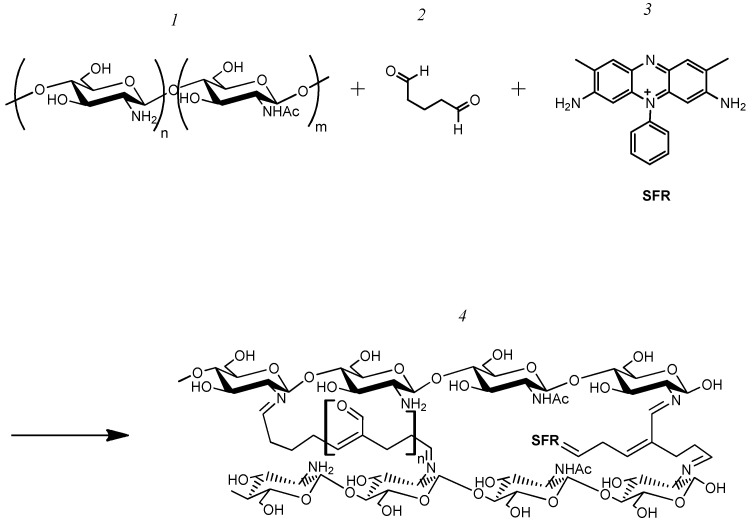
Scheme of the synthesis of a redox-active polymer based on CHIT-SFR, where 1—chitosan; 2—glutaraldehyde; 3—safranin O; and 4—CHIT–SFR redox-active polymer.

A total of 500 µL of a 1% chitosan solution in 1% acetic acid, 5 µL of a saturated solution of one of the electron transport mediators (neutral red—NR, safranin O—SFR, and phenosafranin—FEN), 50 µL phosphate buffer with a pH of 6.8 and 7.5 µL of glutaraldehyde were added. The redox-active polymer was dropped to a GP electrode at an amount of 10 µL and allowed to dry completely. The reaction scheme is illustrated in Figure 2.

Formation of a graphite-paste electrode modified with a redox-active polymer based on BSA (BSA-NR; BSA-SFR; BSA-FEN electrodes).

**Figure 3:**
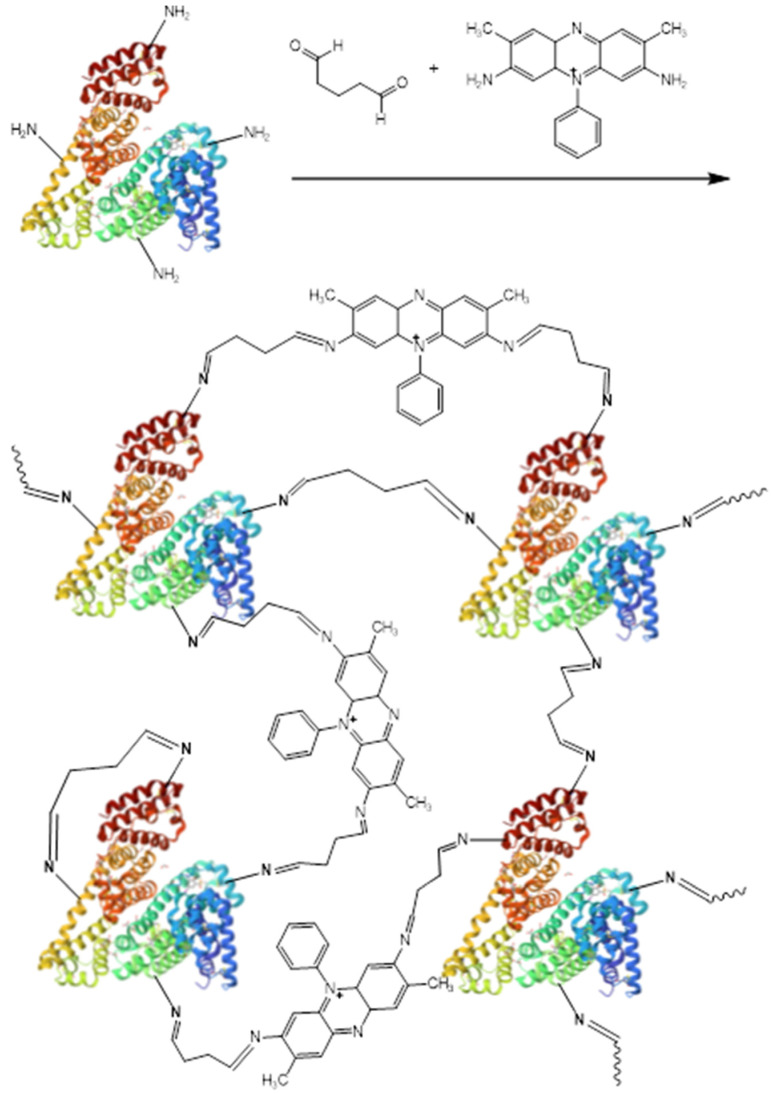
Scheme of the synthesis of a redox-active polymer based on BSA-SFR.

A total of 3.5 μg of BSA, 5 µL of a saturated solution of one of the electron transport mediators (NR, SFR, and FEN), and 50 µL of phosphate buffer with a pH of 6.8 were added. The resulting mixture was shaken for 5 min. Then 7.5 μL of glutaraldehyde was added to the solution and shaken for 30 s. The resulting polymer was dropped to a GP electrode at an amount of 10 µL and allowed to dry completely. The reaction scheme is illustrated in Figure 3.

Formation of a graphite-paste electrode modified with a redox-active polymer based on BSA and ferrocene (BSA-FC electrode).

A total of 0.5 g of BSA was dissolved in 5 mL of buffer solution; 0.05 g of ferrocene carboxaldehyde was dissolved in 5 mL of acetone and added to the BSA solution. The resulting solution was adjusted to a pH of 9.3 by adding 5% K_2_CO_3_. The reaction was allowed to continue for one hour at a temperature of 30 °C. Then, 0.01 g of NaBH_4_ was added to the mixture. After stirring the resulting mixture for ten minutes using a magnetic stirrer, it was kept at room temperature for six hours. The pH of the remaining mixture was adjusted to 6.5 in order to decompose any remaining NaBH_4_ that may have formed. Next, the pH was adjusted to 8.5 by slowly adding a 0.1 M sodium hydroxide solution. Finally, the mixture was centrifuged at 4000× *g* for 20 min. The supernatant resulting from the centrifugation was then dialyzed against a phosphate buffer for several days at a temperature of 4 °C in order to separate any unreacted ferrocene carboxaldehyde from the solution.

**Figure 4:**
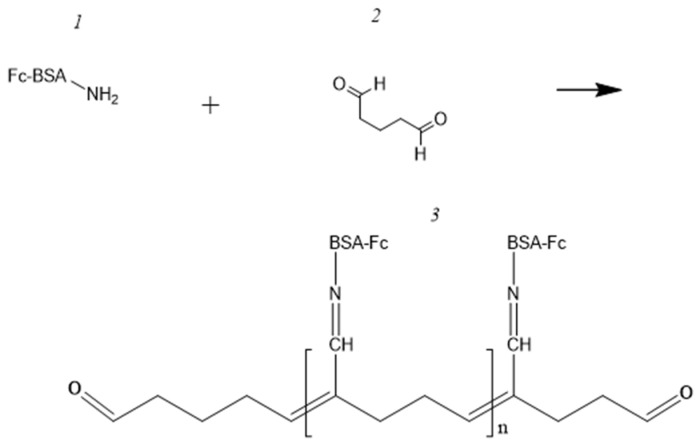
Modification of a redox-active polymer with glutaraldehyde, where 1—modified BSA, 2—glutaraldehyde, and 3—water-insoluble redox-active polymer BSA-FC.

To form the electrode, 0.0035 g of a synthesized polymer was dissolved in 50 mL of phosphate buffer (pH 6.8), and 7.5 μL of glutaraldehyde was added. Moreover, 10 μL of the resulting solution was applied to a GP electrode and allowed to dry completely. The reaction scheme is illustrated in Figure 4.

Formation of a graphite-paste electrode modified with a redox-active polymer based on chitosan and ferrocenecarboxaldehyde (CHIT-FC electrode).

A total of 0.1 g of chitosan was dissolved in 10 mL of 3% acetic acid and a solution of 0.01 g of ferrocenecarboxaldehyde in 7 mL of acetone was mixed for 24 h. Then 0.0530 g of NaBH_4_ was added by stirring. The reaction mixture was stirred for 24 h. The modified polymer was precipitated with a solution of 0.0050 M NaOH to a pH of 10.0. The precipitated polymer was centrifuged, passed through a dialysis membrane, and then dried in an oven at 20 °C for 2 h.

**Figure 5:**
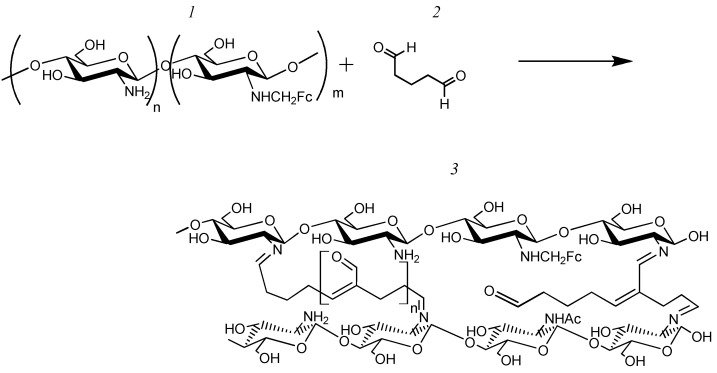
Scheme of the synthesis of a network redox-active polymer based on chitosan and ferrocenecarboxaldehyde (CHIT-FC), where 1 denotes linear modified Chitosan, 2—glutaraldehyde, and 3—networked redox-active polymer CHIT-FC.

To form the electrode, 0.0025 g of the resulting polymer was dissolved in 250 μL of 1% acetic acid. Moreover, 7.5 μL of glutaraldehyde was added to 50 μL of the resulting solution; 10 μL of the resulting mixture was applied to the GP electrode and allowed to dry completely. The reaction scheme is illustrated in Figure 5.

Formation of graphite-paste electrodes modified with a composite material based on redox polymers and nanomaterials ((BSA-NR-CNT/COOH; BSA-NR-CNT/CONH_2_; BSA-SFR-CNT/COOH; BSA-SFR-CNT/CONH_2_; BSA-FEN-CNT/COOH; BSA-FEN-CNT/CONH_2_; BSA-FC-CNT/COOH; BSA-FC-CNT/CONH_2_; CHIT-FC-CNT/COOH; CHIT-FC-CNT/CONH_2_ electrodes).

The surface of the GP electrode was modified with a suspension of multi-walled carboxylated carbon nanotubes CNTs or amidated CNTs with a titer of 50 µg/µL. Then the electrode modified with nanomaterials was dried and a redox-active polymer was added.

Formation of Bioelectrodes.

To create the receptor element for this experiment, 10 µL of a suspension containing 330 mg of wet weight/mL of biomass from the association under investigation—where the number of microorganisms was taken in a ratio of 1:1—was applied onto the electrode. After applying the suspension, the contents of the electrode were fixed with a dialysis membrane.

### 2.6. CV Tests

Cyclic voltammograms were recorded using the Ecotest-VA voltammetric analyzer (Econix-Expert, Moscow, Russia), using a three-electrode system. The working electrode was a modified GP electrode, and the auxiliary electrode was a platinum electrode. A saturated Ag/AgCl electrode was used as the reference electrode. The measurements were performed at a scan rate of 10 mV/s to 200 mV/s at a temperature of 22 °C in a 0.15 M potassium sodium phosphate buffer (pH = 6.8). The cell volume used was 15 mL.

To identify the limiting step in the electron transfer in the “redox-active polymer-electrode” system, the dependence of the limiting current on the logarithm of the scan rate was analyzed. If the limiting step is proportional to the square root of the scan rate (Equation (1)), then the electron transfer is limited by the hopping mechanism. However, if the limiting current is proportional to the scan rate (Equation (2)), then the surface reaction is the limiting factor.

(1) $$I_{p}=-0.496\sqrt{\alpha n}\cdot nFC\sqrt{\frac{FVD}{RT}},$$

where *I_p_*—limiting current; α—transfer coefficient, *n*—number of electrons, *F*—Faraday’s constant, *C*—concentration of electroactive particles, *D*—diffusion coefficient, *R*—gas constant, *T*—temperature, and *V*—scan rate.

(2) $$I_{p}=9.4\cdot 10^{5}\Gamma_{\infty }An^{2}V,$$

where *I_p_*—limiting current; $\Gamma_{\infty }$—limiting number of redox particles on the electrode surface, *A*—electrode surface area, *n*—number of electrons, and *V*—scan rate.

If the limiting step is the surface reaction, then the Laviron equation (Equation (3)) is used to calculate the rate constant for electron transfer from the redox-active polymer to the electrode.

(3) $$\mathrm{logK}=(1-\alpha )\log\alpha +\alpha \log(1-\alpha )-\log\frac{RT}{FnV}-\frac{\alpha \left(1-\alpha \right)nF(E_{a}-E_{K})}{2.3RT},$$

where K—electron transfer rate constant (c^−1^); *α*—transfer coefficient for anode process; (1 − *α*)—transfer coefficient for cathode process; *V*—scan rate; *R*—gas constant; *T*—temperature; *n*—number of electrons; *E_a_*—limiting potential of anode process; and *E_K_*—limiting potential of cathode process.

Cyclic voltammograms for bioelectrodes were recorded before and after the addition of glucose in order to determine the rate constant for the interaction between the enzyme systems of microorganisms and the redox sites on the polymers (Equation (4)).

(4) $$k_{in}=\frac{n\cdot F\cdot {\left(tg\alpha \right)}^{2}}{\left[E\right]\cdot R\cdot T},$$

where *k_in_*—the rate constant for the interaction between the enzyme systems of microorganisms and the redox sites on the polymers; *n*—number of electrons; *F*—Faraday’s constant; *tgα*—The tangent of the slope of the linear dependence between the ratio of limiting anodic currents in the presence and absence of a substrate, and the reciprocal of scan speed square root; *R*—gas constant; *T*—temperature; and [E]—cell titer.

### 2.7. Impedance Spectroscopy

Impedance characteristics were measured using a CS310M potentiostat (Corrtest Instruments, Wuhan, China). For the measurements, a three-electrode measurement circuit was used, where the working electrode was a modified GP electrode, and the auxiliary electrode was a platinum plate with an area of 30 mm^2^. A silver chloride electrode was used as a reference electrode. Impedance measurements were carried out at a voltage of +350 mV (vs. Ag/AgCl) in the frequency range from 40 kHz to 0.02 Hz with a voltage modulation amplitude of 10 mV. Measurements were carried out in a 25 mM potassium phosphate buffer solution (pH 6.8) containing 10 mM of sodium chloride.

### 2.8. Electron Microscopy

A thin layer of platinum–carbon mixture was deposited to the matrix samples using a JEE-4X vacuum deposition unit (JEOL, Tokyo, Japan). The samples were then analyzed using a JSM-6510 LV scanning electron microscope (JEOL, Tokyo, Japan) in a high vacuum mode while recording secondary electrons.

### 2.9. IR Spectroscopy 

The IR spectra of the starting materials and reaction products were recorded using an FMS 1201 infrared Fourier spectrometer (Monitoring, Saint Petersburg, Russia). To measure the spectra of solids, KBr disks were used: a sample of a solid (1–3 mg) was thoroughly mixed with spectroscopically pure potassium bromide (150–200 mg) in a mortar, and the mixture was pressed at a pressure of 7.5–10 ton/cm^2^ for 2–5 min. The resulting sample spectrum was recorded relative to air.

### 2.10. Raman Spectroscopy 

Raman spectra were recorded by an M532 Raman microscope (Spektr-M, Chernogolovka, Russia) using a He–Ne laser with excitation radiation at a wavelength of 532 nm.

### 2.11. Biosensor Measurements 

**Figure 6:**
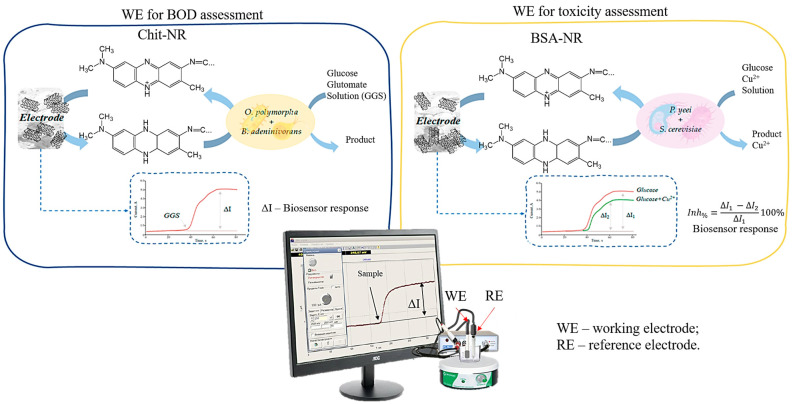
BOD toxicity biosensor design. Insert: the current increasing is associated with the adding of a model solution into the system (GGS for BOD analysis, Glucose and Cu^2+^ solution is for toxicity analysis). For the water sample BOD assessment, the GGS solution is replaced with the sample. For toxicity assessment, Cu^2+^ is replaced with the sample (glucose was diluted in the sample at the same concentration for the control solution test).

To record the signal from the biosensor system, the IPC Micro galvano-potentiostat (Volta, Saint Petersburg, Russia) was used, and dependence of current on time was recorded. The electrodes were connected to the electrochemical detector. The measurements were carried out at a potential of 0.25 V (Figure 6).

The measurements took place in a two-electrode system that was immersed in a measuring cuvette of 5 cm^3^ containing potassium–sodium phosphate buffer. The pH of the buffer was 6.8. The reference electrode—saturated silver chloride—was used. The measurements took place with stirring, using a magnetic stirrer, at a temperature of 20 °C and cell volume of 5 cm^3^. After a stable current level was established, a solution containing the analyte was added to the cell, and the oxidate activity of the microbe was calculated as the current amplitude. After each measurement, the electrochemical cell was washed with a buffer solution.

Rapid assessment of BOD using a biosensor.

To estimate BOD, a bioelectrode was calibrated with a model solution containing glucose and glutamic acid. The BOD_5_ of this solution is 205 mg/L. Taking into account dilution, BOD_5_ for the test samples was determined using a calibration plot pre-constructed on the day of analysis.

Rapid assessment of the toxicity index using a biosensor. 

After establishing a stable current level, 500 μL of 0.1 M glucose solution was added to the cell and the current amplitude was recorded. Next, a 500 μL mixture containing a model toxicant solution or a sample with 0.1 M glucose was added to the cell and the current amplitude was recorded. After each measurement, the cells were washed with a potassium–sodium phosphate buffer solution. Consequently, the current amplitude significantly decreased due to the toxicant’s harmful effects on microbial metabolic activity. The analytical signal is defined as the inhibition index, which is calculated using Equation (5).

(5) $${Inh}_{\%}=\frac{∆I_{glucose}-∆I_{glucose+toxicant}}{∆I_{glucose}}100\%$$

where *Inh*_%_—inhibition index; Δ*I*_glucose−current_ amplitude after added glucose solution; and Δ*I*_glucose+toxicant_—current amplitude after the added mixture containing the sample/toxicant and glucose.

### 2.12. Reference Luminescence Method for Assessing the Toxicity of Water Samples

An Ekolyum-11 test kit (NERA-S, Moscow, Russia) was used for reference testing, which is based on genetically modified *E. coli* bacteria K12 TG1 that have the lux operon from soil-luminous bacteria Photorhabdus luminescens ZM1. “Ekolum-11” was brought to working condition; lyophilized bacteria were rehydrated to prepare a suspension with 1.4 × 10⁷ cells/mL. The density of the bacterial suspensions was determined turbidimetrically at a wavelength of 670 nm and estimated content pre-plotted calibration curves. The luminescence intensity of the bacteria was measured using a Biotox-10M luminometer NERA-S, Russia), and the toxicity index of the water samples was calculated by the luminometer’s program [24].

### 2.13. Reference Chlorella Method for Determining the Toxicity of Water Samples

Chlorella vulgaris was cultivated in 50% of Tamiya media [25]. The final concentration of inoculum was adjusted to an optical density of 0.130 ± 0.006 at 525 nm. Moreover, 48 mL of water samples diluted in Tamiya media were inoculated with 2 mL of algae (experimental group) under aseptic conditions. An identical procedure was followed for inoculation into a control container (Tamiya media without actual samples) (control group), including incubation for 24 h. The optical density of test samples was then measured, and the rate of algal growth inhibition was calculated based on Equation (6).

(6) $${Inh}_{\%}=\frac{A_{control}-A_{experimental}}{A_{control}}100\%$$

where *Inh*_%_—inhibition index; *A*_control_ is the average optical density of the control experiment; and *A*_experimental_ is the average optical density of the analyzed sample.

### 2.14. Determination of BOD Using the Standard Method 

The standard method was used as the reference method for determining BOD_5_ [26]. The dissolved oxygen content was determined using EXPERT-001-4.0.1 (Econix-Expert, Russia).

## 3. Results and Discussion

### 3.1. Formation of Time-Stable Associations among Microorganisms for Rapid Assessment of Toxicity and BOD

Based on our previous research on the formation of receptor systems using monocultures of microorganisms for toxicity assessment [6,7], and biochemical oxygen demand (BOD) [14] under conditions involving a bioelectrocatalytic mediator, and taking into account the growth parameter characteristics, associations among microorganisms for BOD and toxicity assessment were formed (Table S1). To assess toxicity, we proposed forming the following associations: *P. yeei* and *E. coli*, *E. coli* and *S. cerevisiae*, and *S. cerevisiae*, and *P. yeei*. The use of these microorganisms would allow the creation of receptor systems that are sensitive to both heavy metals and organic toxins, thereby enhancing the capabilities of the analytical system. Additionally, the similarity in growth parameters and specific growth rates between these pairs of microorganisms ensures the stability of association composition during the functioning of receptor systems. For the rapid assessment of BOD, the following microbial associations were formed: *O. polymorpha* + *B. adeninivorans*, *P. yeei* + *E. coli*, and *B. adeninivorans* + *P. yeei*. The electrochemical reaction with ferrocene as a model electron transport mediator does not depend on pH, and this mediator has previously been used to assess toxicity when using individual strains of microorganisms [7]. These associations can interact with ferrocene and have been confirmed by biosensor responses at a working potential of 250 mV.

**Figure 7:**
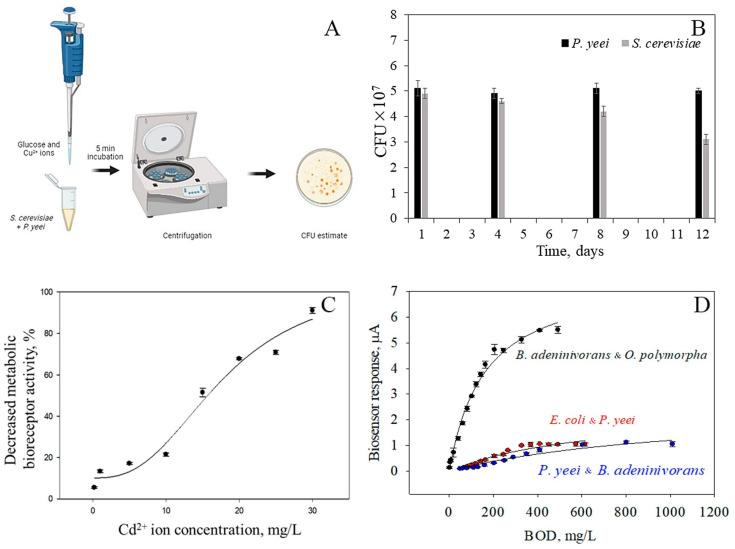
Formation of receptor elements for assessing toxicity and BOD_5_. (**A**) Experimental design for assessing the stability of the associations. (**B**) Results of the CFU determination after *S. cerevisiae* and *P. yeei* formation and toxicity assessment of Cu^2+^ ion as a model toxicant. (**C**) Dependence of the analytical signal or decrease in metabolic activity on the concentration of a model toxicant of a bioreceptor element based on the mediator ferrocene and the association of the bacteria *P. yeei* and yeast *S. cerevisiae* in the presence of Cd^2+^ ions. (**D**) Dependence of the association-based biosensor response on the BOD_5_ of the model sample.

The receptor element for toxicity assessment based on *E. coli* + *S. cerevisiae* showed low stability; the relative standard deviation of the analytical signal exceeded 25% for 7 tests. Systems based on the associations *P. yeei* + *E. coli* and *S. cerevisiae* + *P. yeei* were more stable: the standard deviation of the analytical signal was not more than 10% for 14 measurements. The stability of the composition of formed associations was determined by changing the ratio of the number of cells through seeding on solid media using the Koch method in operating conditions of the receptor elements (experimental scheme shown in Figure 7A). Cu^2+^ ions were used as model toxicants (model toxicants for the ciliate test organism [27]), and the stability of associations was similarly studied to assess BOD_5_. A glucose–glutamic acid solution (1:1) with a total concentration of 3 g/L was used as the model substrate.

When using bioreceptor elements based on the bacteria *P. yeei* and yeast *S. cerevisiae*, there is a change in the ration composition of the association (Figure 7B). By the end of the measurement period, the *P. yeei* bacteria became the dominant species, which may be due to their higher resistance to the action of the toxicant model (Table S1). The association based on *E. coli* bacteria and *P. yeei* is more stable, with the composition not differing significantly from the original 1:1 ratio (Figure S1A). This is due to similar growth parameters for the microorganisms used, including lag phase and exponential growth [7]. However, in the presence of the toxicant, there is a significant reduction in the number of viable microorganisms, which may affect the sensitivity of the bioreceptor element. From Figure S1B, it can be seen that the ratio of microorganisms in associations of *O. polymorpha* and *B. adeninivorans*, as well as *E. coli* and *P. yeei* (Figure S1B), is similar to the original ratio of 1:1, and this microorganism has similar growth parameters. The composition of the association between *B. adeninivorans* and *P. yeei* (Figure S1D) changes quickly: due to their higher specific growth rates, the number of *P. yeei* bacteria increases, and on the 6th day of the experiment, the ratio of *B. adeninivorans* to *P. yeei* microorganisms is 2 to 3. Changes in the composition of the mixture may also be caused by competition, as bacteria actively suppress the growth of the yeast by producing inhibitory compounds or consuming important nutrients. Secretion of antibacterial substances is not the only way bacteria can overcome competitors in a mixed association. Due to their mobility, bacteria can cover yeast cells and become the dominant culture in the mixture [28].

The sensitivity of the receptor elements for assessing toxicity was measured by the concentration of model toxicants that caused a 50% decrease in the activity of the receptor element based on the dependence of the analytical signal of the biosensor on the concentration of the toxicant. The solutions of Cu^2+^ ions (a model toxicant for the test organism ciliates [27]), Cd^2+^ ions (model toxicant for chlorella alga test organism [25]), and a phenol (model toxicant for duckweed test organism [29]) were used as toxicants. The results are presented in Table 1. Receptor elements based on associations of *P. yeei* + *E. coli* or *S. cerevisiae* + *P. yeei* are not inferior to receptor elements based on individual organisms that make up these associations. The receptor element based on the *P. yeei* + *E. coli* association is more sensitive than the receptor based on the individual microorganisms. *P. yeei* + *E. coli* association has the highest sensitivity to Cd^2+^ ions among other biosensors, and the *S. cerevisiae* + *P. yeei*-based receptor element has the highest sensitivity to phenol solutions. For bioreceptors formed to measure BOD_5_ (biochemical oxygen demand), calibration dependencies of the analytical signal on BOD_5_ were obtained using a model solution composed of glucose and glutamic acid, with final concentrations of 150 mg/L for each component. The BOD_5_ value for this solution was 205 mgO_2_/L (Figure 7D) [26,30].

Long-term stability is characterized by the duration of the biosensor’s operation. The long-term stability of the receptor elements for toxicant assessment was determined by measuring the extent of microbial inhibition; the biosensor’s response was measured daily at a model toxicant concentration that caused a 20% decrease in the respiratory activity of the microorganisms (IC_20_). This specific concentration of model toxicants was selected because it allows for a well-defined analytical signal and reduces the load on the receptor element during long-term measurements. The electrode was stored between measurements in a phosphate buffer solution with a pH of 6.8. If the biosensor response decreased by less than 20%, it was considered not suitable for use. The long-term stability of the receptor elements was evaluated in a similar manner to assess BOD_5_ based on the association: the response of the biosensor was measured daily when a solution of glucose and glutamic acid was added to the measuring cuvette with final concentrations of 150 mg/L of each component. The BOD_5_ value of this solution was 205 mgO_2_/L, between measurements, the electrodes were stored in a phosphate buffer solution (pH = 6.8). A comparison of the long-term stability of a biosensor based on association and the individual microorganisms from which the association is composed is presented in Figure S2.

The operational stability of the biosensors was evaluated by the value of the relative standard deviation (RSD) during repeated measurements (15 times) on a standard sample (with a confidence level of 95%). From the data presented in Table 1, it follows that the receptors for assessing BOD_5_ and toxicity allow for a stable analytical signal to be obtained. The reproducibility of the receptors was assessed by calculating the RSD of responses from seven electrodes when a model solution was applied (with a 95% probability of confidence). Based on the obtained results, the developed systems are reproducible, with the RSD not exceeding 10%. The selectivity of the developed systems for BOD_5_ assessment was assessed by evaluating the substrate specificity of the microorganisms in the formed associations. The number of oxidized substrates is presented in Table 1, while the dynamics of substrate oxidation during the operation of the receiving element are shown in Figure S2.

The association between the bacteria *P. yeei* and the yeast *S. cerevisiae* has the greatest potential for toxicity assessment. This combination of microorganisms allows for a more stable analytical signal, with operational stability of about 7%, and more reproducible analytical systems, with reproducibility of 6–7%. In contrast, the association between *P. yeei* and *E. coli* shows less stability in the presence of organic toxicant phenol. Furthermore, the association between the bacteria *P. yeei* and yeast *S. cerevisiae* also has the highest sensitivity to organic toxicant phenol, as at a concentration of 1.6 mg/L of this substance in a sample, and the oxidative activity of the receptor element decreases by 50% (Table 1). The receptor system based on yeasts *O. polymorpha* and *B. adeninivorans* also has great biotechnological potential in terms of BOD_5_ assessment. The stability of the composition, high sensitivity, and ability to analyze within MPC make this a promising option for this purpose (Table 1). It should be noted that the lower limits of the determined concentrations are somewhat inferior to those of bioreceptor elements based on individual cultures. However, this disadvantage can be overcome through the use of nanocomposites. Due to its stable composition and the absence of competition between microorganisms, a receptor element based on the formed association is preferable to a bioreceptor based on activated sludge. This is because the standardization process for the formation of analytical systems can be simplified.

### 3.2. The Formation and Study of Chemical, Spatial Structures, and Electrochemical Properties of Redox-Active Polymers/Composites

**Figure 8:**
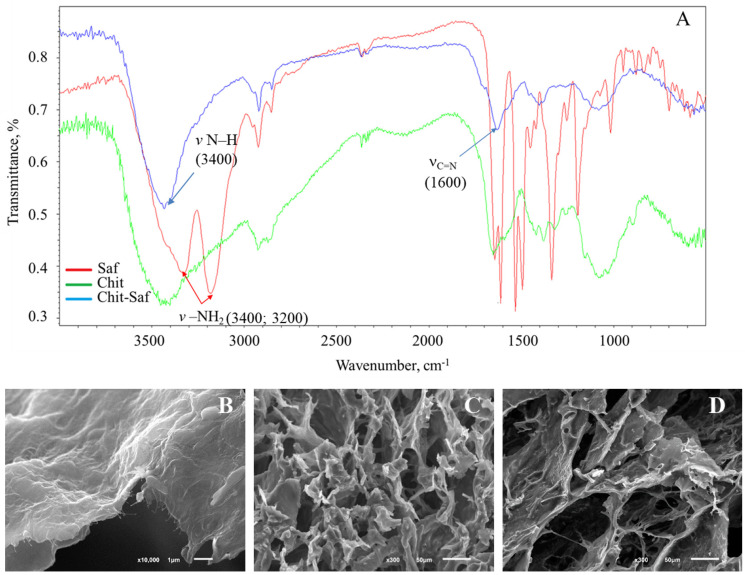
Redox-active polymer for association immobilization. (**A**) IR spectra of safranin O, chitosan, and a redox-active polymer, where v –NH_2_ are stretching vibrations of –NH_2_, v C=N are stretching vibrations of C=N. (**B**) SEM images of BSA-FC-CNT/COOH nanocomposite. (**C**) SEM images of CHIT-FC redox-active polymer. (**D**) SEM images of BSA-FC redox-active polymer.

In order to improve the characteristics of biosensor systems, it is most advantageous to use composite materials based on these redox-active polymers and nanomaterials. To synthesize redox-active polymers, bovine serum albumin (BSA) and chitosan were used as base polymers. These polymers have high biocompatibility, biodegradability, and non-toxicity. These properties are essential for the immobilization of microorganisms. In order to create a conductive structure, the polymers were modified with redox compounds, such as neutral red, safranin O, phenosafranin, and ferrocene carboxaldehyde. Glutaraldehyde was used to create the network structure for the redox-active polymer. The chemical structure of these redox-active polymers was analyzed through infrared spectroscopy. Infrared spectra of chitosan, safranine O, and redox polymers based on these compounds are shown in Figure 8.

In the IR spectra of three compounds—neutral red, safranin O, and phenosafranin—there are absorption bands at around 3400 and 3200 cm^−1^ (Figure 8A), corresponding to the stretching vibrations of NH_2_. These bands are changed after redox-polymers are formed and observed as one peak at 3400 cm^−1^. This is consistent with the expected mechanism in which primary amino groups are involved in the formation of these polymers. There is a change at 1600 cm^−1^ because the Schiff base is formed.

The formation of cross-linking using a redox polymer based on bovine serum albumin, as an example, was studied using Raman spectroscopy in the region of 400–4000 cm^−1^. Overall, the Raman spectra for neutral red (Figure S3A) and bovine serum albumin (Figure S3B) are consistent with the literature [37,38]. The redox-active polymer obtained exhibits fluorescent properties (Figure S3C). This observation can be interpreted as a change in the molecular structure of the original substances, leading to the emergence of new energy levels that interact with external radiation. These results indirectly confirm the formation of a redox-active polymer. Therefore, Raman spectroscopy is a powerful tool for analyzing the structural and electronic properties of substances, even in cases where their fluorescence is not directly observed.

The mass fraction of ferrocene carboxaldehyde in redox-active polymers was determined using atomic absorption spectrometry methods (AAS). The content of neutral red, phenosafranin, and safranine O was determined using a spectrophotometric method (SM): unreacted redox compounds were separated from the polymer using dialysis, and the concentration of redox compounds in the final solution was determined. The mass fraction of redox compounds in the resulting polymers is shown in Table S2.

Redox-active polymers based on neutral red, phenosafranin, and safranin O exhibit an irregular structure, and the formation of dimeric and oligomeric fragments may occur, for example, “BSA-FEN-FEN”, “BSA-BSA”, as well as fragments that do not bind to the polymer, i.e., “FEN-FEN”. 

The architecture of the resulting polymers was examined using scanning electron microscopy. The pore size of these redox-active polymers ranges from 20 to 30 μm (Figure 8C,D). The network structure of these pores allows for the immobilization of a group of microorganisms to form receptor elements.

**Figure 9:**
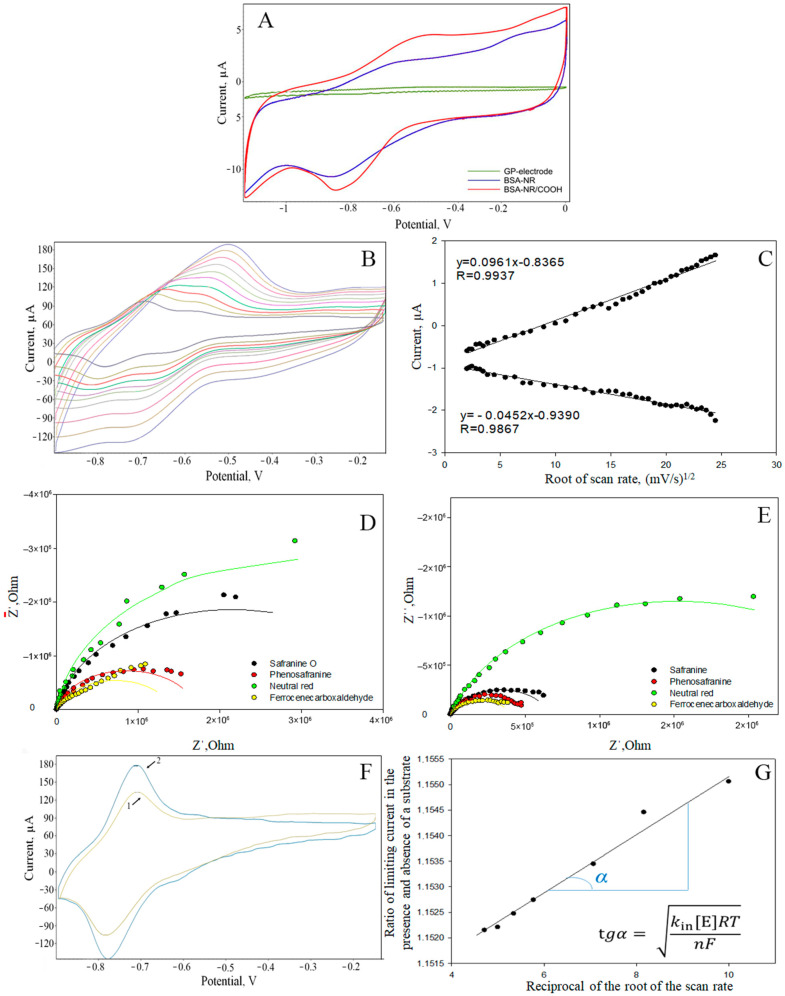
The electrochemical properties of the redox-active polymers and their nanocomposites. (**A**) CV curves of GP-electrode; BSA-NR redox-active polymer; BSA-NR-CNT/COOH nanocomposite at 100 mV/s. (**B**) CV curves of the BSA-NR redox-active polymer for a range of scan rates from 10 to 100 mV/s. (**C**) Determination of the limiting stages of the electronic process in the redox-active polymer BSA-NR. (**D**) Impedance spectroscopy of the studied redox-active polymers based on BSA. (**E**) Impedance spectroscopy of the studied redox-active polymers based on CHIT. (**F**) Cyclic voltammograms were recorded before (line 1) and after (line 2) the addition of glucose in order to determine the rate constant for the interaction between enzyme systems at 10 mV/s. (**G**) Dependence of the ratio of limiting currents in the presence and absence of a substrate on the reciprocal value of the root of the scan rate.

The incorporation of a redox polymer occurs through physical interactions with the polymer backbone (Figure 8B). The electrochemical properties of these redox-active polymers were then studied using cyclic voltammetry and impedance spectroscopy, as shown in Figure 8. The redox-active polymer obtained by cross-linking with neutral red particles has conductive properties, in contrast to the cross-linked BSA without the addition of redox particles (Figure 9A). At potentials of −0.9 V and −0.6 V, cathodic and anodic reactions occur with the participation of redox-active, covalently bound neutral red. (Figure 9A).

The process of electron transfer during the use of redox-active polymers may be limited by either the transfer of electrons between the redox particles in the polymer (the hopping mechanism) or by the transfer of electrons from the redox particles onto the electrode surface (the surface reaction) [39]. The limiting step was determined by examining the dependence of the limiting current on the scan rate (Figure 9B,C). The established nature of the limiting step and the rate constant for heterogeneous electron transfer to the electrode for the redox polymers/composites under study are presented in Table 2. Among redox polymers based on the azine series, those based on phenosafranin are characterized by a faster rate of electron exchange with the electrode surface. The rate constant for the studied polymer is higher than for polymers based on BSA and neutral red as well as those based on chitosan and neutral red.

The electrochemical properties of the resulting redox-active polymers were investigated using impedance spectroscopy. By measuring the impedance characteristics of these systems, the frequency dependencies of the real and imaginary parts of the impedance were determined; these data are presented in the form of Nyquist plots (Figure 9D,E). Based on these plots, the charge transfer resistances of the redox polymers were calculated (Table 2).

To interpret the impedance spectra obtained, the standard Randles equivalent circuit model was used, which includes charge transfer resistance (Rp), ohmic resistance (Rs), and double-layer capacitance (Cdl). A semicircle at high frequencies in the Nyquist plot indicates electron transfer limitation, and its diameter corresponds to Rct. Based on the diagrams obtained (Figure 9D,F), chitosan-based redox-active polymers exhibit significantly lower charge transfer resistances. These polymers interact more efficiently with the electrode, resulting in a larger contact surface and facilitating the flow of electricity through conductive pathways. The lowest Rct value is characteristic of polymers based on FC. When carbon nanotubes and redox-active polymers are used together, the Rct value compared to redox polymers decreases significantly (by two orders of magnitude), indicating an increase in the conductivity of the electrode surface. This can be explained by the excellent conductive properties of aminated and carboxylated nanotubes. Using cyclic voltammetry, it has been shown that when nanomaterials are added, higher electron transfer rates can be achieved (Table 2). However, the use of aminated nanotubes appears to be less effective. The electron-withdrawing properties of the carboxyl groups in the nanomaterial likely play a role in the process of electron transfer. For further research, it would be more promising to explore the use of carboxylated nanotubes in forming receptor systems. While the addition of nanomaterials into most of the composites studied has been successful, the limiting step in this process is often the surface reaction. Nanomaterials increase the rate of electron transfer in a hopping process. However, when using safranin O-based composites, the nature of the limiting step changes compared to using redox-active polymers without nanomaterials. Therefore, carboxylated nanotube-based composites were selected for further study.

### 3.3. Immobilization of Microorganisms into Redox-Active Polymers and Their Nanocomposites

The kinetics of the bioelectrochemical interaction between enzyme systems of microorganisms, such as the yeast *S. cerevisiae* and the bacteria *P. yeei*, which were selected in Section 3.1, were studied. These microorganisms were selected as the basis for the bioreceptor elements of the biosensor used for toxicity assessment. Additionally, the yeast *B. adeninivorans* and *O. polymorpha* were selected in Section 3.1, as they were used as the bioreceptor BOD. The cyclic voltammetry method was used to determine the rate constant for the interaction between the enzyme systems of microorganisms and the redox sites on the polymers [40]. For this purpose, voltammograms were recorded both before and after the addition of glucose. Due to the electrocatalytic oxidation of the substrate, there was an increase in anodic current (Figure 9F,G). The method for determining the rate constant involves fulfilling two conditions. Firstly, it is necessary to use an excess concentration of the substrate. Secondly, the recorded current must be limited by diffusion. The diffusion nature of this limiting stage was confirmed by the linear relationship between the limiting anodic current and the square root of the sweep rate.

In all the studied bioelectrode systems, diffusion was the limiting stage. Assuming that the enzyme systems of microorganisms were in a reduced state due to the high concentration of the substrate, the rate constant for the interaction between the enzyme systems and the studied mediators was determined (Table S3).

Based on the interaction constants, composite materials have been selected for the immobilization of the associations of the yeast species *O. polymorpha* and *B. adeninivorans*, with the CHIT-NR-CNT/COOH nanocomposite, and the association of *S. cerevisiae* and *P. yeei*, with the BSA-NR-CNT/COOH nanocomposite. In these systems, the maximum rate of interaction between enzyme systems from microorganisms and the electroactive sites on redox polymers has been achieved (Table S3). It should be noted that these composite materials transfer electrons onto the electrode an order of magnitude more quickly than other azine composites (Table 2).

To develop bioreceptor elements, we selected conditions under which the current generated by the biosensor was maximum. Based on the obtained operating parameters, methods can be developed to create stable, reproducible, and highly sensitive receptor systems of a universal biosensor for assessing BOD and toxicity (Table S4, Figure S4).

### 3.4. Analytical and Metrological Parameters for the Development of Biosensors for the Rapid Assessment of BOD and Toxicity

To quantify the BOD index in a sample, a bioreceptor element was used based on the association of the yeast species *O. polymorpha* and *B. adeninivorans*, immobilized in a CHIT-NR-CNT/COOH nanocomposite material. Calibration dependencies of the analytical signal on the BOD index were obtained with a solution of glucose and glutamic acid with a known BOD value. The dependence of the analytical signal from the receptor element on the BOD had the form of a hyperbole. The linear range was observed for BOD values between 0.6 and 20 mg/L. The minimum BOD value that could be detected using the receptor element was 0.6 mg/L, and the relative standard deviation of the analytical signal was less than 30%. This allowed for the analysis of samples in natural water with a low BOD index. In addition, the sensor has a wide range of concentration capabilities, which allows for the determination of BOD_5_ indices in contaminated environments without the need to dilute samples. The main analytical and metrological parameters of a bioreceptor element, based on the association of the yeast species *O. polymorpha* and *B. adeninivorans* immobilized in a composite material, were compared to BOD analyzers from reported biosensors and commercial analyzers (Table 3).

The use of a composite material based on carbon nanotubes and a redox polymer modified with neutral red chitosan has increased the efficiency of electron transfer, making it possible to increase the sensitivity of BOD determination (Table 3). In terms of sensitivity, the developed receptor element is slightly inferior to certain analogs, but the value of the lower limit at 0.6 mg/dm^3^ makes it possible to analyze samples with BOD values below the maximum allowable concentration (MAC). Unlike these models [15], the developed bioreceptor is characterized by a wider concentration range, allowing samples to be analyzed without needing to be diluted. The obtained characteristics are close to those of a biosensor based on activated sludge, but the developed system has a more stable composition (long-term stability is 34 days and operation stability or relative standard deviation of the analytical signal 15 consecutive measurements is 6%), further simplifying the production of these receptors. In terms of the duration of a single measurement, the development analytical system exceeds that of many commercial analogs, while being more compact.

The sensitivity of a bioreceptor element based on *S. cerevisiae* and *P. yeei*, immobilized in a BSA-NR-CNT/COOH nanocomposite, was assessed to determine the toxicity. The assessment was carried out using model toxicants, such as Cu^2+^ (a model toxicant for ciliates [27]), Cd^2+^ (for Chlorella [25]), Zn^2+^ (luminescent bacteria) [24], phenol (duckweed) [29], p-nitrophenol, Pb^2+^, and Ni^2+^ ions. For each toxicant, a dependence of the analytical signal was obtained, which is the reduction in oxidative activity of microorganisms on the concentration of the toxicant. From this, the values of the toxicant concentration, which causes a 50% decrease in activity, were determined (Table S5). The relative standard deviation of the analytical signal for 15 consecutive measurements is 10% and long-term stability is 7 days.

It should be noted that the developed receptor system based on *P. yeei* and *S. cerevisiae* outperforms similar biosensors in terms of sensitivity to Ni^2+^, Pb^2+,^ and Cd^2+^ ions, as well as phenol. The developed system could become an alternative to using biosensors based on luminescent bacteria. In terms of sensitivity, it is only second to that of Zn^2+^ ion analysis. Standard test objects (such as Daphnia, Lemna, and Chlorella vulgaris), as well as commercial analyzers that are based on these (e.g., IPS-03 and Biotester-2M), are more sensitive than the developed bioreceptor system, but rapid assessment of aquatic environment quality on the same day of sampling is feasible using the developed element. This is because a single analysis takes only 10 min. MAC are much lower than those of test objects. However, the long analysis time for biotesting when evaluating toxicity limits the wider use of toxicology methods in environmental monitoring due to the high analysis times. The developed bioreceptor for assessing toxicity enables rapid analysis of the condition of the object being studied, complementing the findings of physicochemical analysis methods.

### 3.5. Testing of the Developed Receptor Elements for Rapid Assessment of Toxicity and Biochemical Oxygen Consumption on Natural Water Samples

To assess the potential of using the developed receptor elements for determining the BOD_5_ index and toxicity of surface waters, samples were collected from various locations in the Tula Region and the Stupino Urban District. For the study, samples were obtained from rivers, lakes, reservoirs, ponds, streams, and a quarry. The results of the analysis are presented in Table S6.

The results of BOD determination using the standard method and the developed receptor element are statistically indistinguishable (Fisher’s test, modified student’s *t*-test, and Welch’s approximate *t*-test were applied to the data). Regarding toxicity assessment, a difference was found in the results of the biosensor analysis compared to the results of the standard method for three samples. Samples taken from the Karachevsky Pond (Kimovsk) and Kantorsky Pond (Simonovo Village) showed different toxicity classes. This is due to the different sensitivities of microorganisms in the receptor element and luminescent bacteria. As a result, there is an error in assessing the toxicity class of the reservoir, whose toxicity index is on the border between non-toxic and toxic. The BOD value of the sample from Kremnitsa River (Stupinsky District) exceeds the MAC and a difference in the toxicological assessment results compared to the values of the standard method and the developed receptor system was observed. Additional toxicity tests were performed on this sample using a more sensitive *Chlorella* test organism [25]. The results obtained correlated with those from the biosensor analysis, and the sample was classified as toxic. Therefore, to create a universal biosensor, receptor elements have been developed that allow for the express monitoring of water quality in terms of BOD and the assessment of the toxicity of a sample.

## 4. Conclusions

In this work, we developed receptor elements for the rapid diagnosis of surface water conditions based on two indicators: biochemical oxygen demand and toxicity. Cyclic voltammetry and impedance spectroscopy were used to study electrochemical processes in nanocomposites, showing the potential of a composite made of neutral red and carboxylated nanotubes for immobilizing associations. The heterogeneous electron transfer rate constants for CHIT-NR-CNT/COOH and BSA-NR-CNT/COOH composites were 0.77 ± 0.06 and 0.89 ± 0.03 s^−1^, respectively. The charge transfer resistances were (10.6 ± 0.3)∙10^5^ and (4.5 ± 0.3)∙10^5^ Ohm. Furthermore, the interaction between covalently attached neutral red in chitosan-based composites and yeast enzyme systems was investigated. It was found that *O. polymorpha* had a reaction rate constant of 110 ± 10 L/g × s, while *B. adeninivorans* had a rate constant of 66 ± 2 L/g × s. In the BSA-NR-CNT/COOH composite, the yeast *S. cerevisiae* and bacteria *P. yeei* had reaction rates constant of 72 ± 6 and 120 ± 20 L/g × s, respectively. It was revealed that the developed receptors are not inferior to known laboratory models and commercial analyzers: the sensitivity of the receptors based on the immobilization of the yeasts *O. polymorpha* and *B. adeninivorans* in a composite allowed the analysis of BOD within the MAC for up to 34 days and the time for a single analysis was 4–5 min. The receptor based on the association between *P. yeei* and *S. cerevisiae* is superior in its sensitivity to Ni^2+^, Pb^2+,^ and Cd^2+^ ions as well as phenol compared to analogous biosensors. This system can be an alternative to using commercial biosensors based on luminescent bacteria (Biotox-10M), and a single analysis time of 10 min allows the rapid assessment of environmental conditions. The developed receptor elements were tested on samples from surface waters in the Tula region and Stupino district: rivers, lakes, reservoirs, ponds, streams, and a quarry. The high correlation between the obtained BOD values and the toxicity of the samples, as measured by two different methods, allows for the rapid analysis and assessment of these parameters using a new type of equipment. 

## Figures and Tables

**Table 1:** Characteristics of receptor elements formed based on the associations among microorganisms for assessing the toxicity and BOD_5_.

Mediator Biosensors for Toxicity Assessment										
Biomaterial/ Mediator	Concentrations of Toxic Substances (IC_50_), Causing a 50% Decrease in the Activity of a Receptor Element, mg/L			Long-Term Stability, Days		Operational Stability, %		Reproducibility, %		Ref.
	Cu^2+^	Cd^2+^	Phenol	Cu^2+^	Phenol	Cu^2+^	Phenol	Cu^2+^	Phenol	
Association of *S. cerevisiae* and *P. yeei*/ferrocene	15.9	15.6	1.6	5	5	7.5	7.0	5.6	6.7	This work
Association of *E. coli* and *P. yeei*/ferrocene	23.8	7.5	8.1	4	4	7.3	10.3	5.2	9.6	[7]
*S. cerevisiae*/ferrocene	2.7	17.5	1.8	5	5	6.9	11.5	4.9	5.1	[7]
*E. coli*/ferrocene	47.6	8.9	17.6	3	3	6.92	10.81	7	9	[7]
*P. yeei*/ferrocene	21.1	18.2	9.9	9	10	4.9	5.3	4.5	4.8	[6]
Activated sludge/K_3_[Fe(CN)_6_]	19.8	13.4	-*	-	-	-	-	-	-	[31]
*S. cerevisiae*/menadione and K_3_[Fe(CN)_6_]	10.1	13.9	44.5	-	-	-	-	-	-	[32]
*E. coli*/thionine	20.2	36.2	-	-	-	-	-	-	-	[23]
*E. coli*, *B. subtilis*, *S. cerevisiae*/p-benzoquinone	16.5	20.5	-	-	-	-	-	-	-	[33]
**Mediator Biosensors for BOD_5_ Assessment**										
**Biomaterial/** **Mediator**	**Number of Oxidized Substrates, Pcs.**			**Long-Term Stability, Days**				**Linear Range BOD_5_, mg/L**		**Ref.**
*P. yeei*, *E. coli*/ferrocene	16			20				61–164		This work
*O. polymorpha*, *B. adeninivorans*/ferrocene	16			19				2–140		This work
*P. yeei*, *B. adeninivorans*/ferrocene	12			5				49–290		This work
*S. cerevisiae*/menadione and K_3_[Fe(CN)_6_]	-*			-*				10–220		[34]
*E. coli*/ferrocene	7			17				0.7–1.59		[15]
*O. polymorpha*/ferrocene	14			9				-*		[15]
*B. adeninivorans*/ferrocene	16			5				2.5–21		[15]
*P. yeei*/ferrocene	22			22				1.3–200		[35]
*Chromobacterium violaceum*/K_3_[Fe(CN)_6_]	-*			-*				20–225		[35,36]

-* not detectable.

**Table 2:** Electrochemical properties of the obtained redox-active polymers and their nanocomposites.

Redox-Active Polymers/Nanocomposite	Limiting Stages of the Electronic Process	Rate Constant for Heterogeneous Electron Transfer to the Electrode, s^−1^	Charge-Transfer Resistance, 10^5^ Ohm
BSA-FEN	Surface reaction	0.32 ± 0.02	17.4 ± 0.4
BSA-FEN-CNT/COOH	Surface reaction	0.42 ± 0.02	75 ± 2
BSA-FEN-CNT/CONH_2_	Surface reaction	0.36 ± 0.02	93 ± 4
CHIT-FEN	Surface reaction	0.34 ± 0.03	4.83 ± 0.06
CHIT-FEN-CNT/COOH	Surface reaction	0.54 ± 0.03	24 ± 4
CHIT-FEN-CNT/CONH_2_	Surface reaction	0.41 ± 0.02	89 ± 4
BSA-SFR	Surface reaction	0.26 ± 0.02	57 ± 3
BSA-SFR-CNT/COOH	Hopping mechanism	-	121 ± 3
BSA-SFR-CNT/CONH_2_	Hopping mechanism	-	243 ± 8
CHIT-SFR	Surface reaction	0.23 ± 0.02	6.7 ± 0.1
CHIT-SFR-CNT/COOH	Surface reaction	0.57 ± 0.02	19 ± 1
CHIT-SFR-CNT/CONH_2_	Surface reaction	0.54 ± 0.05	41 ± 4
BSA-NR	Surface reaction	0.0119 ± 0.0006	72 ± 2
BSA-NR-CNT/COOH	Surface reaction	0.89 ± 0.03	4.5 ± 0.3
BSA-NR-CNT/CONH_2_	Surface reaction	0.64 ± 0.05	14 ± 1
CHIT-NR	Hopping mechanism	-	30.5 ± 0.5
CHIT-NR-CNT/COOH	Surface reaction	0.77 ± 0.06	10.6 ± 0.3
CHIT-NR-CNT/CONH_2_	Surface reaction	0.44 ± 0.03	57 ± 3
BSA-FC	Surface reaction	0.45 ± 0.01	14 ± 1
BSA-FC-CNT/COOH	Surface reaction	0.87 ± 0.04	9.3 ± 0.1
BSA-FC-CNT/CONH_2_	Surface reaction	0.63 ± 0.09	19 ± 2
CHIT-FC	Surface reaction	0.44 ± 0.02	4.10 ±0.05
CHIT-FC-CNT/COOH	Surface reaction	0.86 ± 0.04	7.1 ± 0.2
CHIT-FC-CNT/CONH_2_	Surface reaction	0.56 ± 0.08	22 ± 5

**Table 3:** Main characteristics of BOD analyzers.

Biomaterial/Immobilization ^1^	Redox Compound	Parameters ^2^	Ref.
Association of *O. polymorpha* and *B. adeninivorans*/CHIT-NR-CNT/COOH	Neutral red, covalently bonded to chitosan	R: 0.6—20 mg/L S: 22 compound L: 34 days T: 4–5 min	This work
Association of *O. polymorpha* and *B. adeninivorans*/D	Ferrocene in graphite paste	R: 61–164 mg/L O: 6% S: 16 compound L: 19 days T: 4–5 min	This work
*B. adeninivorans*/D	Ferrocene in graphite paste	R: 2.5–21 mg/L S: 16 compound L: 5 days T: 4–5 min	[15]
*Saccharomyces cerevisiae*/BC-CB	Solution of potassium hexacyanoferrate(III) and menadione	R: 10–220 mg/L T: 5 min	[35]
*Activated sludge*/AC-G	Methylene blue	R: 1–100 mg/L L: 65 min	[41]
Biox-1010 analyzer	-	R: 5–100,000 mg/L T: 3–15 min	[5]
BioMonitor analyzer	-	R: 1–200,000 mg/L T: 3–4 min	[5]

^1^ immobilization: CHIT-NR-CNT/COOH—composite based on carbon nanotubes and chitosan modified with neutral red; CHIT-FC-CNT—composite based on carbon nanotubes and ferrocene-modified chitosan; D—encapsulation in dialysis membrane; BC-CB—inclusion in a composite based on bacterial cellulose and carbon black inclusion in a composite based on bacterial cellulose and carbon black; AC-G—composite based on graphene and chitosan and albumin cryogel. ^2^ Parameters: R—БПK range; S—the number of oxidized substrates. L—long-term stability; T—measurement time for a single sample. O—operation stability, %.

## Data Availability

The original contributions presented in the study are included in the article and Supplementary Material, further inquiries can be directed to the corresponding authors.

## Supplementary Materials

The following supporting information can be downloaded at https://www.mdpi.com/article/10.3390/polym16101431/s1, Figure S1: The study of the stability of the compositions of associations: A. *E. coli* and *P. yeei* in the presence of Cu^2+^ ions; B. *O. polymorpha* and *B. adeninivorans*; C. *E. coli* and *P. yeei*; D. *B. adeninivorans* and *P. yeei*; Figure S2: Long-term stability of a bioreceptor element based on A *P. yeei* bacteria; B. *E. coli* bacteria; C. associations of *E. coli* and *P. yeei* bacteria. The horizontal line indicates a 50% drop in biosensor response from the maximum value. D. Change in substrate specificity of the bioelectrode due to association of bacteria; Figure S3: Raman spectra: A. bovine serum albumin, B. Neutral red, C. Redox-active polymer based on bovine serum albumin and neutral red. λo = 532 nm. Figure S4: Effect of pH on the biosensor response: A. *O. polymorpha* and *B. adeninivorans* association; B. *S. cerevisiae* and *P. yeei* association. Table S1: Initial data for forming associations. Table S2: Mass fraction of redox-active particles in polymers. Table S3: The rate constants for the interaction of enzyme systems of microorganisms with electroactive sites of the redox polymer. Table S4: Operating parameters of bioreceptor elements. Table S5: Sensitivity to model toxicant solutions, expressed in toxicant concentrations (IC_50_) causing a 50% decrease in receptor element activity. Table S6: Results of analysis of natural waters. References [42,43,44,45,46,47,48,49,50,51,52,53,54] are cited in the Supplementary Materials.
