# Supplementary material for: A “2-in-1” Bioanalytical System Based on Nanocomposite Conductive Polymers for Early Detection of Surface Water Pollution
Source: Polymers (Basel). 2024 May 17;16(10):1431. doi: 10.3390/polym16101431 (PMC11125136; doi:10.3390/polym16101431)
Supplement: Supplementary file 1 [file polymers-16-01431-s001.zip › polymers-2982463-supplementary.pdf]

polymers

MDPI

Supplementary Materials

A "2-in-1" Bioanalytical System Based on Nanocomposite Conductive Polymers for Early Detection of Surface Water Pollution

Anna S. Kharkova [1], Anastasia S. Medvedeva [1], Lyubov S. Kuznetsova [1], Maria M. Gertsen [2], Vladimir V. Kolesov [3], Vyacheslav A. Arlyapov [1,*] and Anatoly N. Reshetilov [4]

1 The Research Center «BioChemTech», Tula State University, 300012 Tula, Russia; anyuta_zaytseva@mail.ru (A.S.K.); ilyuhina.nastya@mail.ru (A.S.M.); l.s.latunina@gmail.com (L.S.K.)

2 Laboratory of Soil Chemistry and Ecology, Tula State Lev Tolstoy Pedagogical University, , 300026 Tula, Russia; gertsen@tsput.ru;

3 Kotelnikov Institute of Radioengineering and Electronics (IRE) of Russian Academy of Sciences, 111250 Moscow, Russia; kvv@cplire.ru

4 Federal Research Center «Pushchino Scientific Center for Biological Research of the Russian Academy of Sciences», G.K. Skryabin Institute of Biochemistry and Physiology of Microorganisms, Russian Academy of Sciences, 142290 Pushchino, Russia; anatol@ibpm.pushchino.ru

* Correspondence: v.a.arlyapov@tsu.tula.ru

Table of contents

Formation of Time-Stable Associations of Microorganisms for Rapid Assessment of Toxicity and BOD. 2

The formation and study of chemical, spatial structures, and electrochemical properties of redox-active polymers / composites. 5

Immobilization of microorganism into redox-active polymers and their nanocomposites. 6

Analytical and metrological parameters for the development of biosensors for the rapid assessment of BOD and toxicity. 7

Formation of Time-Stable Associations of Microorganisms for Rapid Assessment of Toxicity and BOD.

The following microorganisms were used to form an association in order to assess toxicity: bacteria *Escherichia coli* K802 (*E. coli*), *Paracoccus yeei* VKM B-3302 (*P. yeei*), *Pseudomonas veronii* DSM 11331^T (*P. veronii*), and yeast *Saccharomyces cerevisiae* VKM Y-1173 (*S. cerevisiae*), *Ogateae polymorpha* VKM Y-2559 (*O. polymorpha*). These microorganisms are quite sensitive to heavy metals and organic compounds, as they naturally live in nature water. *E. coli* bacteria have been used as a testing object in biotest methods [19]. They react acutely to the presence of heavy metals in solutions and are therefore widely used in the creation of biosensors to determine the overall toxicity of aquatic ecosystems. *P. yeei* has been used to analyze the toxicity of aqueous extracts from perfumery and cosmetics products, as well as the total amount of bio-degradable organic substances present in water [42]. Bacteria *P. veronii* were previously isolated from activated sludge by the authors of this work. These microorganisms are able to decompose various aromatic compounds and adsorb heavy metal ions from wastewater [43]. Therefore, bacteria are used to clean soils and wastewater of various pollutants, as well as to monitor the state of the environment. The yeast *S. cerevisiae* is used for biosorption of heavy metals [44] and in the development of biosensors to assess the content of copper ions [45] and analyze the toxicity of water. The oxidative activity of yeast *O. polymorpha* is greatly influenced by the presence of heavy metals in a sample [46].

To create a receptor element based on the associations of microorganisms, the yeast *Ogataea polymorpha* VKM Y-2559 (*O. polymorpha*), the yeast *Blastobotrys adeninivorans* VKM Y-2677 (*B. adeninivorans*), the bacterium *Escherichia coli* K-802 (*E. coli)*, and the bacterium *Paracoccus yeei* VKM B-3302 (*P. yeei*) was selected. The yeasts, *O. polymorpha* and *S. cerevisiae*, and *B. adeninivorans*, are capable of oxidizing a wide range of organic compounds [47, 48]. The *B. adeninivorans* is used as a production crop in hydrolysis plants and is characterized by its high salt tolerance [49]. Yeast *O. polymorpha* is actively used in biotechnology for the proteins production. In analytical biotechnology, they are used to create biosensors for the detection of methyl and ethyl alcohol. Depending on growth conditions, they can change their specificity and can be used to create specialized receptors for certain effluents [46]. The yeast *S. cerevisiae* is also known for its use in food and beverages and widely used to form mediator-based BOD biosensors [50]. *E. coli* bacteria can be found in natural and wastewater, and they are one of the components of the standard analysis methods, which increases the sensitivity of determining BOD_5 when they are included in the receptor element [51]. The bacteria *P. yeei* and *P. veronii*, which are isolated from activated sludge, can oxidize a wide range of organic compounds under the conditions of mediator electrocatalysis.

Table S1. Initial data for forming associations.

Growth phase		*P. yeei*	*E. coli*	*P. veronii*	*B. adeninivorans*	*S. cerevisiae*	*O. polymorpha*
Specific growth rate, h^{-1}		0.6±0.2	0.6±0.1	0.26±0.03	0.18±0.02	0,71±0,04	0.17±0.02
**Sensitivity of the method**		**A mediator toxicity biosensor based on these microorganisms and FC**					
Concentrations of model toxicant solutions (IC_{50}) causing a 50% decrease in the	Cu^{2+}	21.1	47.6	286	-*	2.7	162
	Cd^{2+}	18.2	8.9	164	-	17.5	23
	phenol	9.9	17.6	13.5	-	1.8	10.2

activity of the receptor element.						
**Biosensor characteristics**	**A mediator BOD biosensor based on these microorganisms and FC**					
Number of oxidized substrates, pcs.	22	7	12	16	20	14
Long-term stability, days	22	17	77	5	22	9
Linear range BOD_5, mg/l	1.3	0.7	1.6	2.5	1,5	3.3

*This strain of microorganisms was not used for toxicity assessment, as it is known to be halotolerant [50].

The results of a study on the stability of association composition.

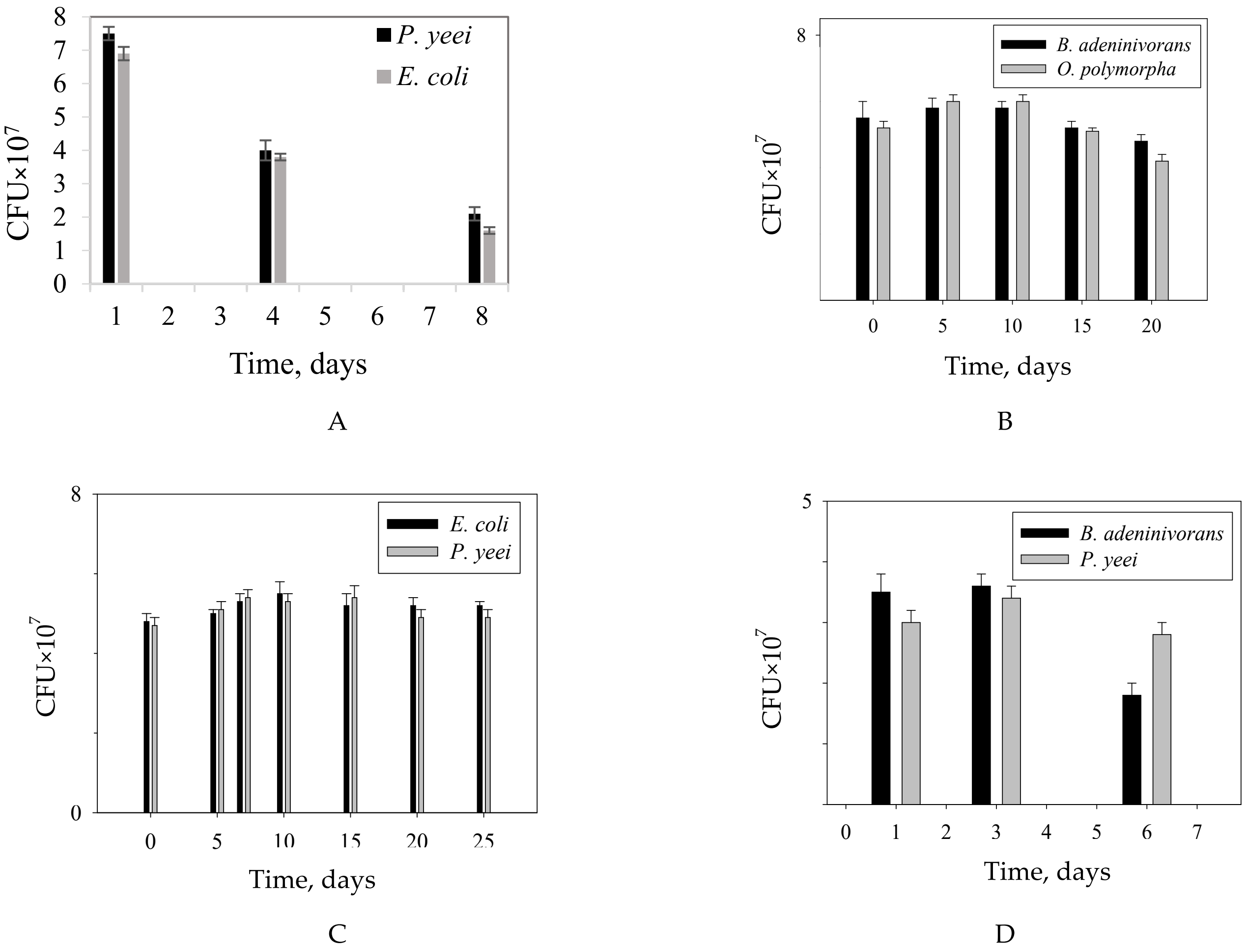

Figure S1. The study of the stability of the compositions of associations: A. E. coli and P. yeei in the presence of Cu^{2+} ions B. O. polymorpha and B. adeninivorans C. E. coli and P. yeei D. B. adeninivorans and P. yeei.

The long-term stability of receptor elements determines the recommended service life. For each combination and the individual cultures from which it was formed, the dynamics of the analytical signals were studied by introducing a model solution containing glucose and glutamic acid. Fig. 2S shows the changes in the long-term stability of bioreceptors based on the combination of *E. coli* and *P. yeei* bacteria and the individual microorganisms that comprise this combination.

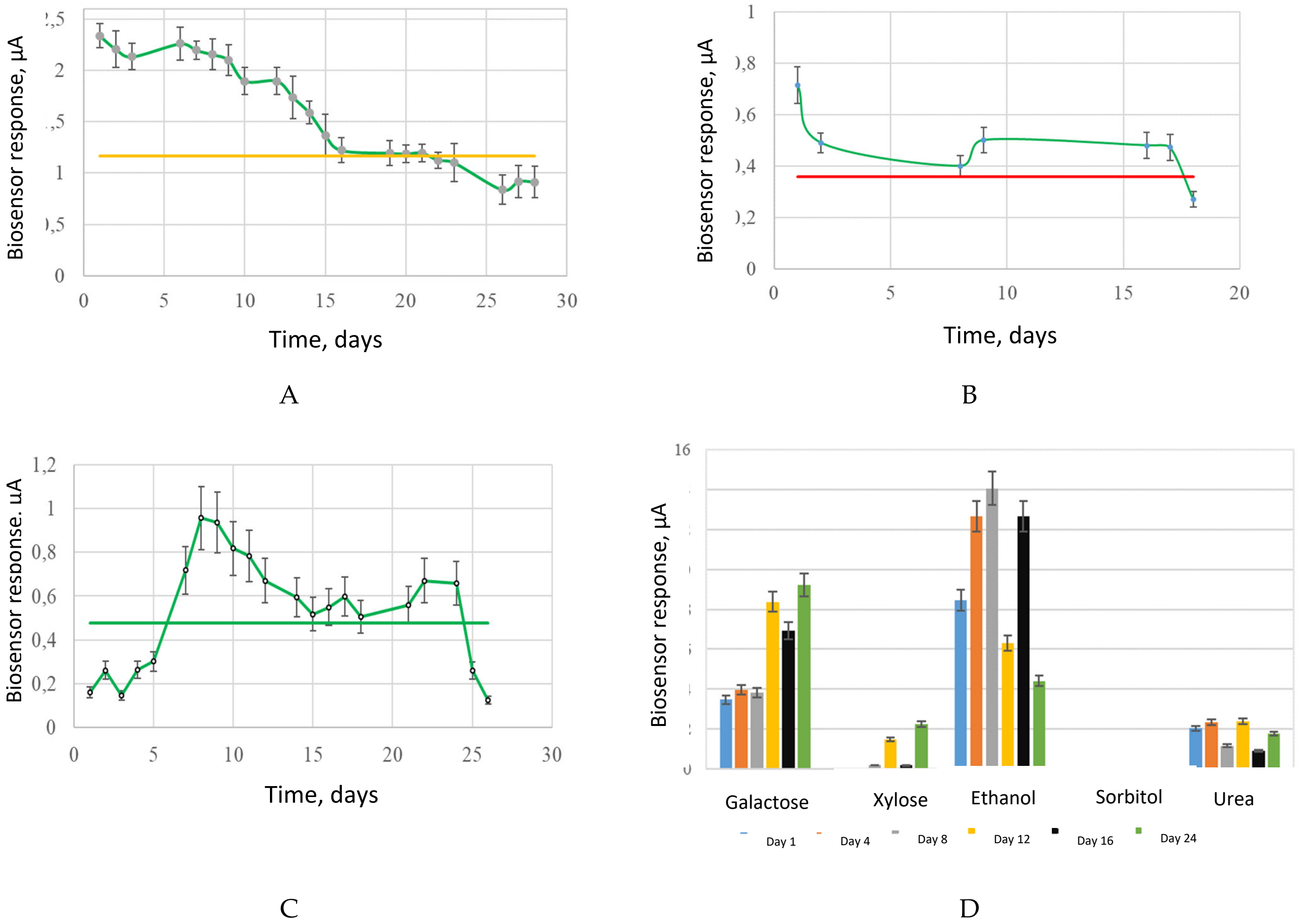

Figure S2. Long-term stability of a bio-receptor element based on A - P. yeei bacteria, B - E. coli bacteria, C - associations of E. coli and P. yeei bacteria. The horizontal line indicates a 50% drop in biosensor response from the maximum value. D - Change in substrate specificity of bioelectrode due to association of bacteria.

The dynamics of the bioreceptor response based on individual cultures gradually decrease, with the microorganisms *P. yeei* dropping by more than 50% on the 22nd day (fig. 2S-A) or the microorganisms *E. coli* dropping by more than 50% on the 18th day (fig. 2S-B). A bioreceptor based on these cultures is characterized by an adaptation period of 5 days (fig. 2S-C), after which a maximum oxidative activity period of the receptor element occurs, and by the 25th day, the biosensor response has decreased by more than 50% from its maximum value. The formed bacterial association maintains its oxidative activity for 20 days within the consortium, but requires 5 days to prepare the electrode for use before the cells within the association have adapted. To create receptor systems that are stable during operation, changes in the substrate specificity of bioelectrodes based on the *P. yeei* and *E. coli* association were studied (fig. 2S-D). Galactose, ethanol, sorbitol, urea, and xylose have been used as substrates in this study. Galactose and xylose are well oxidized by the *P. yeei* strain. Urea is also oxidized, but this association is not sensitive to this substance. Ethanol is oxidized by *E. coli*. Sorbitol is oxidized by both strains. Fig. S2-D shows the dynamics of the bioreceptor response to these different substrates.

Analyzing the dynamics of bioreceptor responses based on the data presented in Fig. 2S-D, from days 1 to 4, it was observed that 3 out of 5 substrates - ethanol, galactose, and urea - elicited a response in the microorganisms, which was apparently associated with their adaptation (Fig. 2S-B). The rate of substrate oxidation decreased in the following order: ethanol > galactose > urea. On day 8, the sensor response reached its maximum value. By this time, the cells in the strains had passed through a period of adaptation (Fig.

2S-B), and a small response was observed for xylose. The ratio of substrate oxidation remained the same as on day 1 of observation: ethanol > galactose > urea > xylose. On day 12, the response of the biosensor decreased, a similar trend was observed during the oxidation of a solution containing glucose and glutamic acid (Fig. 2S-B). In addition, the efficiency of substrate oxidation by the association changed: the greatest response was recorded with galactose. This can be related to a longer adaptation period of *P. yeei*, as they are the organisms responsible for galactose oxidation. Individual microorganisms of the *E. coli* cells do not respond well to galactose. From days 14-24, the activity of the receptor element varied between 50% and 60% of its maximum value (Fig. 2S-C). On day 16, the ratio of effectiveness for oxidized substrates became equal - the greatest response being recorded for ethanol (Figure 2S-D). Presumably, this is due to a decrease in oxidative activity of *P. yeei*. On day 24, the rate of substrate oxidation decreased in the following order: galactose > ethanol > urea > xylose. This may be due to the loss of oxidative activity in the *E. coli* bacteria.

Thus, the dominant role of bacteria in the composition of the association varies: *E. coli* adapts faster as part of the receptor (a greater response to ethanol is observed), but is significantly inferior to *P. yeei* bacteria in long-term stability. Therefore, after the adaptation of *P. yeei* bacteria and at the final stage of functioning of the electrode (when the oxidative activity of the association reaches 50% of the maximum value), the maximum response to galactose is observed. The biosensor still does not respond to sorbitol, indicating competition between two bacterial strains for a specific substrate throughout the entire operating period of the working electrode. Both *P. yeei* and *E. coli* exhibit oxidative activity as a part of the association. This is confirmed by the results of the microbiological study (Fig. 1S-C).

The formation and study of chemical, spatial structures, and electrochemical properties of redox-active polymers / composites.

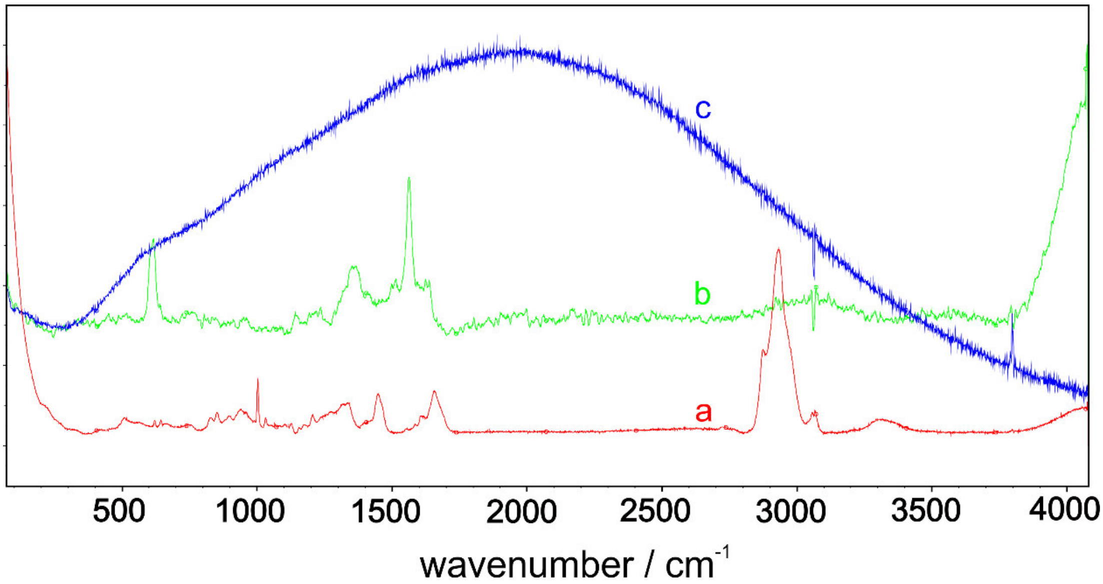

Figure S3. Raman spectra of A. bovine serum albumin, B. neutral red, C. redox-active polymer based on bovine serum albumin and neutral red. λo = 532 nm.

Table S2. Mass fraction of redox-active particles in polymers.

Redox-active polymer	Determination method	Mass fraction, %
BSA-FC	AAS	18.2
CHIT-FC	AAS	9.1

BSA-NR	SM	16
CHIT-NR	SM	4.3
BSA-FEN	SM	4.2
CHIT-FEN	SM	3.4
BSA-SFR	SM	4.3
CHIT-SFR	SM	4.0

It should be noted that, when using BSA as a basis for a redox polymer, a higher number of "BSA-FEN" modifications are formed than when using chitosan (Table S2). However, the number of amine groups through which modifications can occur with redox active particles in BSA is lower than in chitosan. This is likely due to the fact that BSA reacts with ferrocene carboxaldehyde, neutral red, phenosafrainin, and saffrain O in pH > 7. This greatly facilitates the nucleophilic addition reaction and crosslinking chitosan in an alkaline medium is difficult due to its poor solubility.

Immobilization of microorganism into redox-active polymers and their nanocomposites.

Table S3. The rate constants for the interaction of enzyme systems of microorganisms with electroactive sites of the redox polymer.

**Redox-active polymers/nanocomposite**	**The rate constants, l/g×s**			
	O. polymorpha	***B. adeninivorans***	***S. cerevisiae***	***P. yeei***
BSA-FEN	-*	18±0,3	75±4	32±7
BSA-FEN-CNT/COOH	-*	17±5	36±2	46±2
BSA-SFR	-*	-*	23±8	3.1±0.4
BSA-SFR-CNT/COOH	-*	-*	13±8	2.6±0.2
BSA-NR	59.0±0.1	89.7±0.1	72±6	120±20
BSA-NR-CNT/COOH	11.6±0.4	1.0±0.3	130±10	170±30
BSA-FC	56±3	5.6±0.5	43±2	83±4
BSA-FC-CNT/COOH	152±3	51±9	98±9	34±9
CHIT-FC	6.2±0.4	-*	-*	16.7±0.4
CHIT-FC-CNT/COOH	9.6±0.3	-*	-*	23.6±0.3
CHIT-FEN	7.5±0.3	67±2	-*	4±1
CHIT-FEN-CNT/COOH	18.3±0.3	72±4	-*	10±3
CHIT-SFR	-*	-*	7±1	-*
CHIT-SFR-CNT/COOH	-*	-*	7.3±0.7	-*
CHIT-NR	110±10	66±2	210±20	-*
CHIT-NR-CNT/COOH	370±20	320±30	12	93±5

-* In these systems, the interactions between the enzyme systems of microorganisms and the electroactive sites in redox polymers were not observed. The limiting current did not change when glucose was added.

The magnitude of the current generated by the biosensor in the presence of the analyte can vary depending on the operating parameters of the analytical system, such as pH, salt concentration in the buffer solution, concentrations of mediators, weight of carbon nanomaterials, and temperature. For the bioreceptor elements, values for the operating parameters in the biosensors, such as the pH of the working electrolyte, the specific density of biomaterials on the electrode, concentrations of buffer solution salts, concentrations of studied mediators, and temperatures, were selected (Table S4, fig.4S).

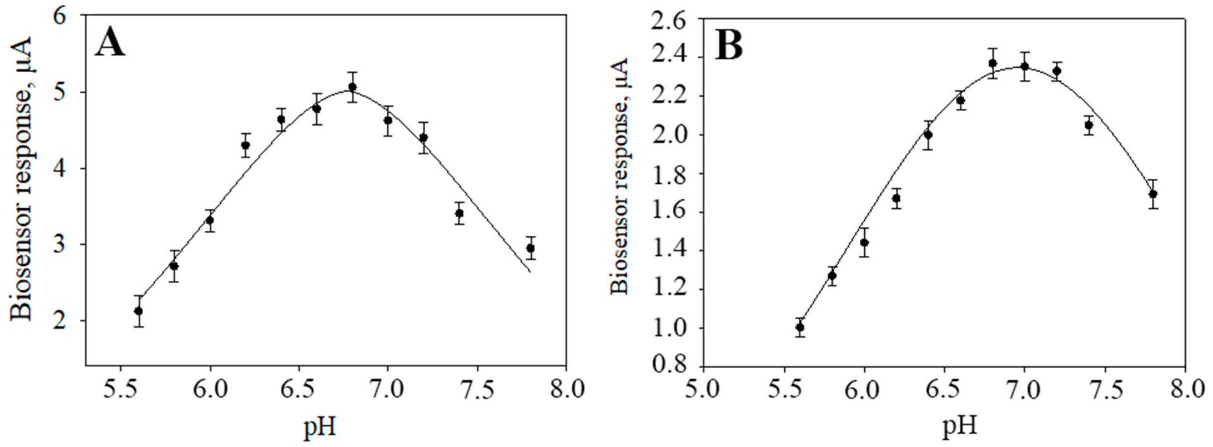

Figure S4. Effect of pH on the biosensor response: A. O. polymorpha and B. adeninivorans association; B. *S. cerevisiae* и P. yeei association.

Table S4. Operating parameters of bioreceptor elements.

Operating parameter	Associations of *O. polymorpha* and *B. adeninivorans*	Associations of *S. cerevisiae* and *P. yeei*
pH	6.8	6.8
Salt concentration in the buffer solution, M	0.080	0.080
Concentrations of mediators, M	5	5
Weight of carbon nanomaterials, μg	500	375
Temperature, C	15-25	15-25

Analytical and metrological parameters for the development of biosensors for the rapid assessment of BOD and toxicity.

The sensitivity of a bioreceptor element based on *S. cerevisiae* and *P. yeei*, immobilized in a BSA-NR-CNT/COOH nanocomposite has been assessed to determine the toxicity. The assessment was carried out using model toxicants, such as Cu^{2+} (a model toxicant for ciliates), Cd^{2+} (for Chlorella), Zn^{2+} (luminescent bacteria), phenol (duckweed), as well as p-nitrophenol, Pb^{2+}, and Ni^{2+} ions. For each toxicant, a dependence of the analytical signal was obtained, which is the reduction in oxidative activity of microor-ganisms on the concentration of the toxicant. From this, the values of the toxicant concen-tration, which causes a 50% decrease in activity, were determined. The sensitivity of the developed bioreceptor element and its analogues for assessing the toxicity of solutions of model toxicants is presented in Table S5.

Table S5. Sensitivity to model toxicant solutions, expressed in toxicant concentrations (IC50) causing a 50% decrease in receptor element activity.

Redox-compound/ bio-material	IC_{50} toxicant, mg/l							Ref.
	Ni^{2+}	Pb^{2+}	Cd^{2+}	Cu^{2+}	Zn^{2+}	Phenol	p-nitrophenol	
BSA-NR-CNT/COOH/ *P. yeei, S. cerevisiae*	9.4	3.2	7.6	8.9	22.1	7.5	5	This work
FC/ *P. yeei*	-*	9.9	18.2	21.1	47.5	9.9	2.1	[52]
p-Benzoquinone/ *E. coli, B. subtilis, S. cerevisiae*	-*	-*	20.5	16.5	-*	-*	-*	[33]

Menadione and potassium hexacyanoferrate(III) / *S. cerevisiae*	17.06	34.6	13.9	10.1	-*	44.5	-*	[34]
Ryaska (Lemna)	1.19	5.5	0.33	0.33	0.9	-*	-*	[8]
Daphnia	0.65	0.82	0.17	0.29	1.8	-*	-*	[8]
Vibrio fischeri	25.2	36.0	52.5	34.4	4.64	-*	-*	[8]
Chlorella vulgaris	-*	0.476	0.301	-*	-*	-*	-*	[53]
Maximum allowable concentration (MAC)	0.01	0.006	0.005	0.001	0.01	0.001	0.01	[54]

Table S6. Results of analysis of natural waters.

**Sampling location**	**BOD Standard Method [24], mg/L**	**BOD biosensor based on O. *polymorpha* and *B. adeninivorans* immobilized in CHIT-NR-CNT/COOH, mgO_2/ L**	**Toxicity Standard Method [26], %**	**Toxicity biosensor based on *P. yeei, S. cerevisiae* immobilized in BSA-NR-CNT/COOH, %**
Khaninsky Pond, Cherepetka River	5±1	3.9±0.4	Non-toxic	Non-toxic
Latinka stream, Kosaya Gora village	6±1	6.1±0.4	Non-toxic	Non-toxic
Goryachka Lake, Kosaya Gora village	6±1	5,7±0,6	Non-toxic	Non-toxic
Upa river, Osinovaya Gora village	5±1	4.8±0.6	Non-toxic	Non-toxic
Pond in Lyubichki village	2.3±0.3	2.4±0.2	Non-toxic	Non-toxic
Upa river, Svoboda village	5±1	4.6±0.4	Non-toxic	Non-toxic
Oka river, Bunyrevo village	5±1	5.9±0.3	Non-toxic	Non-toxic
Tulitsa river, Tula town	5±1	4.2±0.3	Non-toxic	Non-toxic
Novoye Lake, Voronka river	5±1	4.4±0.3	Non-toxic	Non-toxic
Venevka river	5±1	4.2±0.4	Non-toxic	Non-toxic
Gorodilovka stream, Stupino town	2.5±0.6	2.3±0.3	Non-toxic	Non-toxic
Kremnitsa river, Stupinsky district	8±1	8.0±0.7	Non-toxic	Toxic 45±5
Oka river, Stupinsky district	5±1	4.8±0.4	Non-toxic	Non-toxic
Belopesotsky quarry, Stupinsky district	2.5±0.3	2.2±0.3	Non-toxic	Non-toxic
Luzhniki pond, Stupinsky district	1.8±0.5	2.2±0.3	Non-toxic	Non-toxic
Big Pond Museum-Estate Yasnaya Polyana	5±1	5.4±0.4	Non-toxic	Non-toxic
Voronka river, Tula town	4.5±0.6	4,2±0.3	Non-toxic	Non-toxic
Pond in Zheleznya village	2.3±0.3	2.5±0.4	Non-toxic	Non-toxic
Kantorsky Pond, Simonovo village	1.4±0.2	1.5±0.2	Toxic 50±20	Non-toxic
Upa river, Zarechye village, Shchekinsky district	4.0±0.5	4.3±0.6	Non-toxic	Non-toxic

Karachesvsky pond, Kimovsk town	9±1	9.5±0.8	Non-toxic	Toxic<br>23±2
Tulitsa river, Fedyashevo village	3.3±0.1	3.8±0.2	Non-toxic	Non-toxic
Fedyashevka river, Fedyashevo village	3.3±0.7	4.0±0.3	Non-toxic	Non-toxic
Pond in the village of Zhelybino, Tula region (sample 1)	4.9±0.6	5.2±0.3	Non-toxic	Non-toxic
Pond in the village of Zhelybino, Tula region (sample 2)	2.3±0.3	2.5±0.3	Non-toxic	Non-toxic
Platonovsky Pond, Tula town	4.9±0.6	5.1±0.7	Non-toxic	Non-toxic
